# Maternal and child health interventions in Nigeria: a systematic review of published studies from 1990 to 2014

**DOI:** 10.1186/s12889-015-1688-3

**Published:** 2015-04-09

**Authors:** Musa Abubakar Kana, Henry Victor Doctor, Bárbara Peleteiro, Nuno Lunet, Henrique Barros

**Affiliations:** Department of Community Medicine, Faculty of Medicine, Kaduna State University, Kaduna, Nigeria; EPIUnit – Instituto de Saúde Pública da Universidade do Porto (ISPUP), Porto, Portugal; Integrated Programme and Oversight Branch, Division for Operations, United Nations Office on Drugs and Crime, Abuja, Nigeria; Department of Clinical Epidemiology, Predictive Medicine and Public Health, University of Porto Medical School, Porto, Portugal

**Keywords:** Maternal and child health, Interventions, Coverage, Effectiveness, Systematic review, Nigeria

## Abstract

**Background:**

Poor maternal and child health indicators have been reported in Nigeria since the 1990s. Many interventions have been instituted to reverse the trend and ensure that Nigeria is on track to achieve the Millennium Development Goals. This systematic review aims at describing and indirectly measuring the effect of the Maternal, Newborn, and Child Health (MNCH) interventions implemented in Nigeria from 1990 to 2014.

**Methods:**

PubMed and ISI Web of Knowledge were searched from 1990 to April 2014 whereas POPLINE® was searched until 16 February 2015 to identify reports of interventions targeting Maternal, Newborn, and Child Health in Nigeria. Narrative and graphical synthesis was done by integrating the results of extracted studies with trends of maternal mortality ratio (MMR) and under five mortality (U5MR) derived from a joint point regression analysis using Nigeria Demographic and Health Survey data (1990–2013). This was supplemented by document analysis of policies, guidelines and strategies of the Federal Ministry of Health developed for Nigeria during the same period.

**Results:**

We identified 66 eligible studies from 2,662 studies. Three interventions were deployed nationwide and the remainder at the regional level. Multiple study designs were employed in the enrolled studies: pre- and post-intervention or quasi-experimental (n = 40; 61%); clinical trials (n = 6;9%); cohort study or longitudinal evaluation (n = 3;5%); process/output/outcome evaluation (n = 17;26%). The national MMR shows a consistent reduction (Annual Percentage Change (APC) = −3.10%, 95% CI: −5.20 to −1.00 %) with marked decrease in the slope observed in the period with a cluster of published studies (2004–2014). Fifteen intervention studies specifically targeting under-five children were published during the 24 years of observation. A statistically insignificant downward trend in the U5MR was observed (APC = −1.25%, 95% CI: −4.70 to 2.40%) coinciding with publication of most of the studies and development of MNCH policies.

**Conclusions:**

The development of MNCH policies, implementation and publication of interventions corresponds with the downward trend of maternal and child mortality in Nigeria. This systematic review has also shown that more MNCH intervention research and publications of findings is required to generate local and relevant evidence.

**Electronic supplementary material:**

The online version of this article (doi:10.1186/s12889-015-1688-3) contains supplementary material, which is available to authorized users.

## Background

Poor maternal and child health indicators have been a recurring public health challenge in Nigeria since documentation of national Maternal, Newborn, and Child Health (MNCH) statistics began in the early 1990s [1-15]. To address this problem, many interventions were instituted to ensure that Nigeria achieves the relevant Millennium Development Goals (MDGs) [11,16-25]. Nevertheless, various intervention reports have documented mixed findings of the successes and challenges as well as threats to the attainment of MDGs 4 and 5 (child and maternal mortality reduction, respectively) in Nigeria [10,21,22,26-32]. It has been observed that Nigeria is off track in meeting MDG 4 and according to the United Nations mortality estimates, Nigeria has only achieved an average of 1.2% annual reduction in under-five mortality since 1990. And in order to meet MDG 4, Nigeria needed to have achieved an annual reduction rate of 10% in the five years leading to 2015 [10-33].

To design MNCH interventions for post-2015 Nigeria, it will be imperative to provide evidence-based assessment of the previously implemented interventions in terms of volume, coverage, and effectiveness. This will respond to calls for evidence-based decision making and the recognition that better-informed decisions enhance impact and cost-effectiveness [34-38]. Consequently, the application of systematic reviews is essential in strengthening the capacity of institutions to make evidence-based decisions due to its transparent, rigorous, replicable, and timely assessments [35-40].

The primary objective of this systematic review is to identify and synthesize published studies on MNCH interventions in Nigeria from 1990 to 2014. We specifically made a cross-sectional description (trend in time and place) of the interventions with respect to study design and type of intervention as well as target population, coverage and outcome of interventions. The secondary objective was to examine concurrent trends in the development of MNCH policies and publication of intervention studies with maternal, newborn, and under-five mortality using published data to represent rates at different times between 1990 and 2014.

## Methods

### Search strategy

**Figure 1:**
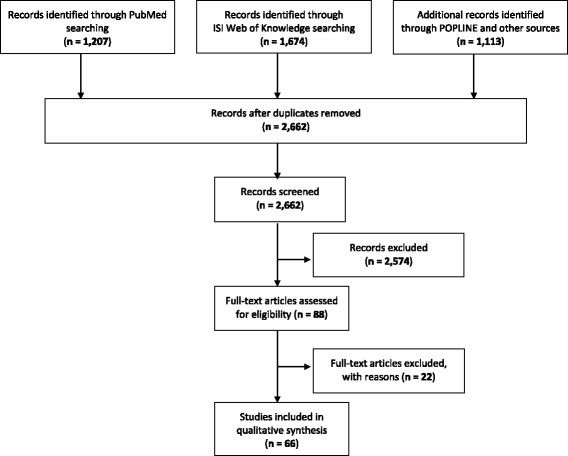
Systematic review flowchart.

PubMed®, ISI Web of Knowledge™ and POPLINE® were searched for articles published from 1 January 1990 to 17 April 2014 (16 February 2015 for POPLINE®) to identify studies reporting interventions targeting women of childbearing age and children in Nigeria from 1990 to 2014. The year 1990 was selected as the starting point because the availability of national level estimates of MNCH indicators started with the 1990 Demographic and Health Survey (DHS) [12,13,15,41,42]. The search expression included synonyms and MeSH terms, which were as follows: (maternal OR child OR newborn) health (Nigeria OR Nigerian) (strategies OR promotion OR intervention OR program OR programme OR rural). An additional step involved the supplemented searches of reference lists of the included articles to assess whether more eligible studies could be identified and included. The search and study selection process is provided in the systematic review flow chart (See Figure 1) and PRISMA checklist (Additional file 1).

### Study inclusion and exclusion criteria

A list of references of published studies was generated and independently screened by two reviewers (MK and HD) in three consecutive steps, by applying pre-defined criteria. First, studies were excluded considering only information presented in the title and abstract. Second, full texts of articles not previously excluded were assessed to determine their eligibility for the review. And finally, full texts were re-evaluated for data extraction.

The search strategy and selection criteria were based on the guidelines for systematic reviews of health promotion and public health interventions [40]. The criteria used to select studies primarily reflected the questions being answered in this review [40,43]. The criteria for exclusion of studies were applied step wise (criterion 1 to 5) on each study and a study was excluded if it did not satisfy the first or a subsequent criterion: (1) **Type of Study**: the non-eligible studies were review articles (except systematic review of interventions), editorial, comment, guidelines and cross-sectional studies; (2) **Intervention**: studies that were non-interventional were ineligible; (3) **Topic of study**: studies with topics not reporting on MNCH were excluded; (4) **Target population**: studies that were not targeting mothers or under-five children in Nigeria were not included; and (5) **Outcome**: studies that were not reporting on MNCH outcome indicators were disqualified. International studies that included Nigeria were covered by the review.

### Data extraction strategy

Disagreements between the independent assessments of the reviewers (MK and HD) were resolved by consensus or after discussion with a third researcher (BP). Two investigators (MK and HD) evaluated independently the selected studies to extract the data on sampling procedures; sample characteristics; rationale of study; type of intervention; sponsorship of intervention; outcome of intervention in terms of maternal health promotion; prevention of obstetric complications; intermediate outcome targeting interventions – contraceptives; child health promotion; prevention and treatment of childhood diseases; and health system strengthening. Differences in the data extracted by the two investigators were discussed until consensus, and involving a third researcher (BP), whenever necessary.

### Data synthesis and presentation

A narrative and graphical synthesis was used to analyze and present findings. The results of data abstraction were summarized in Additional file 2: Table S1, which outlines the type, objective and target of intervention, coverage and measurement of effect for each intervention. We summarize and present the qualitative synthesis of the characteristics of the studies (See Additional file 2: Table S1 for data extraction tool). The result of the systematic review was also integrated into the charts outlining the trend of maternal and under-five mortality. The classification of interventions adopted was according to the level of health care delivery. And there are some interventions that are targeting multiple levels of care because interventions and strategies for improving MNCH are closely related and provided through a continuum of care approach [44]. The findings were also mapped to illustrate the geographical distribution of published studies based on cumulative number of interventions implemented per state using ArcGIS [45].

### Maternal and under-five mortality trends

To monitor trends in maternal and under-five mortality, we derived data from the Nigeria Demographic and Health Surveys (DHS) from 1990 to 2013 [12-42]. Poisson regression analysis was performed using the Joinpoint software [46,47], in order to identify significant changes in the mortality trends (allowing for up to 1 joinpoint). For each of the segments obtained in the best model, the estimated annual percent change (APC) was computed by fitting a regression line to the natural logarithm of the rates using calendar year as a regressor variable (i.e., given y = a + b*x* where y = ln(rate) and *x* = calendar year, the APC is estimated as 100 × (eb-1)).

### Document analysis of national policies, strategies, and guidelines for MNCH

The review of national MNCH policies, strategies, and guidelines was conducted by document analysis of an annotated national bibliography on digitized health policies and guidelines of the Federal Ministry of Health from 1988–2012 [48]. Policies published between 2012 and 2014 were derived by supplementary search. Since policies normally take long to be developed and enacted, additional literature about MNCH policies or health-associated laws (e.g. National Health Act 2014) enacted between 1990 and 2014 were sought from publications of relevant Government institutions available as printed documents and on the Internet, including information websites.

## Results

### Review of the published MNCH interventional studies in Nigeria (1990–2014)

Sixty-six eligible studies were identified from 2,662 studies, after applying pre-defined exclusion criteria to the title/abstract and the full text evaluation [18,20,22,31,49-108]. Additional file 2: Table S1 presents a detailed description of the included studies. The thematic qualitative synthesis of the publications presented below examines the authorship, intervention-publication interval, geographical location, target population, intervention strategy, coverage and their outcomes. Some of the studies did not provide information on one or more of the above themes thus limiting the total number of studies being reported on some thematic features.

### Author reference, publication year, geographical location and setting of intervention

Nigerian nationals as first authors accounted for 52 (79%) of studies and the remainder 14 (21%) had non-Nigerian nationals as first authors. Among the studies included in this review, 52 (79%) were published in international scientific publications, 7 (10.5%) in Nigerian-based journals and 7 (10.5%) were evaluation reports. The frequency of publications between 1990 and 1999 was 8 (12%), in the decade of 2000–2009 was 21 (32%) while the majority 37 (56%) of the included studies were published between 2010 and 2014. The average number of publications per year in the 24 years of observation was 2.75 papers per year. The interval between the completion of intervention and publication ranged from 0–8 years with a mean of 2.57 ± 1 years. There were studies reporting on interventions in a single region 50 (78%), multiple regions 11 (17%) or national coverage 3 (5%). The distribution of implemented intervention studies within Nigeria according to geo-political regions: north-west (10), north-central (3), north-east (3), south-east (12), south-south (6) and south-west (16). There was one study reporting on multiple international sites including Nigeria. Further analysis showed that 20 (33.3%) of these studies were conducted in urban, 32 (53.3%) in rural or eight (13.3%) in both settings. The site of intervention was classified as heath system (n = 5) community based (n = 42) and hospital based (n = 19), which could be first or referral level.

### Implementing organizations, sponsorship, year and duration of implementation

The sponsorship/implementation of the interventions were by Government of Nigeria (n = 9), foreign donors/implementing partners (n = 15) or academic/health institutions (n = 25). Joint sponsorship or implementation partnerships were reported in 15 (22.7%) intervention studies. The Government of Nigeria included its three tiers (federal, state or local) and their resources (financial, human and material) were used to facilitate the implementation of MNCH interventions. The distribution of the duration of implementation were as follows: less than a year (n = 31), 1–2 years (n = 7), 3–5 years (n = 13), 5–9 years (n = 2), 10 years or more (n = 2). The duration of implementation ranged from 1 month to 120 months (10 years) with a mean of 25.1 months (2.1 years).

### Target population, selection criteria and sample size

Women of childbearing age were targeted in 37 (56%) of the included studies, under-five children were the focus in 18 (27%) interventions and both were targeted in 11 (17%) interventions. The guidance for the selection of intervention area was specified by epidemiological factors (e.g. high burden of MNCH problems) in 31 (48%) studies or purposively in 34 (52%). The sample sizes were small population studies of <100 participants (n = 12; or 20%), medium scale of 100–1000 participants (n = 30; 49%) and large population studies of more than 1000 participants (n = 19; 31%).

### Study design, intervention strategies and outcomes

Multiple study designs were employed in the enrolled studies; pre/post-intervention/quasi-experimental (n = 40; 61%), clinical trials (randomized or non-randomized) (n = 6; 9%), cohort study or longitudinal evaluation (n = 3; 5%), process/output/outcome evaluation (n = 17; 26%). Only 23 (35%) of studies had a control or comparison group, while 43 (65%) did not. The range of interventions were targeting adolescents and pre-pregnancy (n = 12; 18%), pregnancy (n = 9; 14%), childbirth (n = 12; 18%), postnatal (mother and newborn) (n = 1; 2%), infancy and childhood (19; 29%), integrated maternal, newborn, and child health (crosscutting community and health system strategies) (n = 13; 19%). The outcomes of the different interventions included maternal health promotion (e.g. utilization of family planning, antenatal care, and prevention of mother to child transmission of HIV) (n = 12; 18%), prevention and management of obstetric complications (e.g. safe management of eclampsia, ante and postpartum heamorrhage, and clinical audit of quality of obstetrics services) (n = 20; 30%), child health promotion (e.g. immunization and infant feeding) (n = 5; 8%); prevention and treatment of childhood diseases (e.g. Insecticide treated net (ITN) use and home management of malaria) (n = 16; 24%), and health system strengthening (e.g. policy for free MNCH services and electronic health information management system) (n = 13; 20%).

### Narrative and graphical synthesis

The multiplicity of intervention designs, strategies and outcomes made quantitative methods, including meta-analysis and effect size for synthesis impossible and inappropriate. Therefore, the published intervention studies were categorized and described for the coverage and indirect measurement of effectiveness as reported in the following narrative and graphical synthesis.

### Trends in maternal mortality in Nigeria, 1990–2013

**Figure 2:**
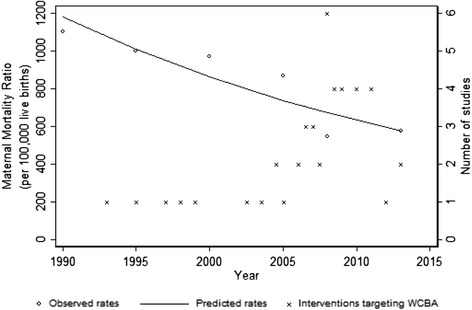
Line graph illustrating the trends in maternal mortality ratio and distribution of maternal health intervention studies in Nigeria, 1990–2013.

Figure 2 illustrates the trend of maternal deaths. The national maternal mortality ratio (deaths per 100,000 live births) shows a consistent reduction during the period of observation (APC = −3.10%, 95% CI: −5.20 to −1.00%) and a marked decrease in the slope is observed in the period that features a cluster of published studies (2004–2014).

### National trend of under-five mortality rate in Nigeria, 1990–2013

**Figure 3:**
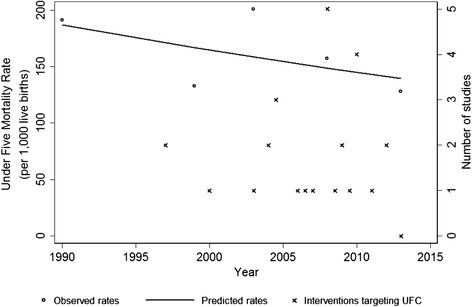
Line graph illustrating the trends of under-five mortality rate and distribution of child health intervention studies in Nigeria, 1990–2013.

Although not statistically significant, a downward trend in the under-five mortality is observed (APC = −1.25%, 95% CI: −4.70 to 2.40%) and coincides with the implementation of most of the studies (Figure 3) during the first decade of the 21st century.

### Regional trend of under-five mortality rate in Nigeria, 1990–2013

**Figure 4:**
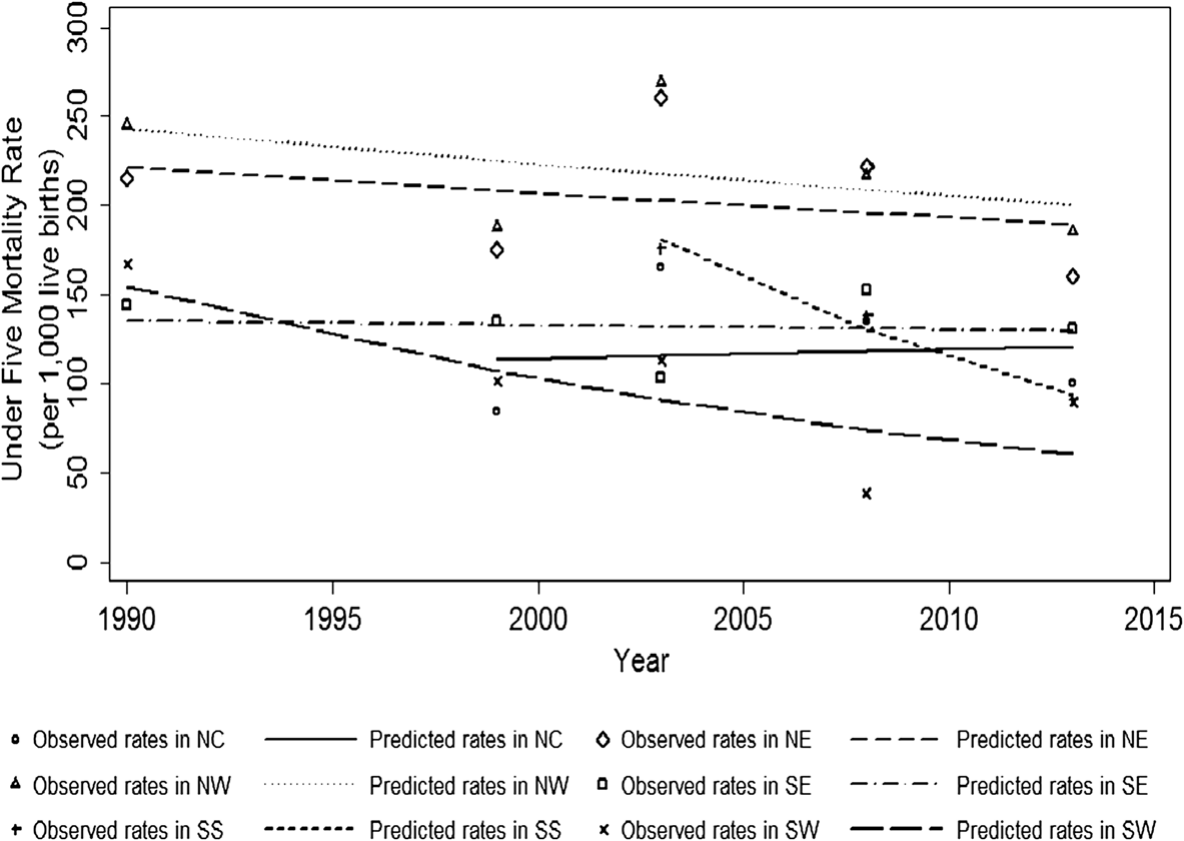
Line graph illustrating the regional trends of under-five mortality rate in Nigeria, 1990–2013.

Despite the decline observed in almost all regions during the period of 1990–2014, regional variation in the rate of decline of under-five mortality rate (U5MR) was observed (Figure 4). North-central region shows a slight increase from 1999 to 2013 (APC = 0.43%, 95% CI: −13.60 to 16.70%), while the highest decline was observed in the South-south region during 2003–2013 (APC = −6.38%, 95% CI: −17.60 to 6.30%). The regions with consistently the highest U5MRs are the north-west and the north-east.

### Geographical coverage of published MNCH interventions in Nigeria, 1990 to 2014

**Figure 5:**
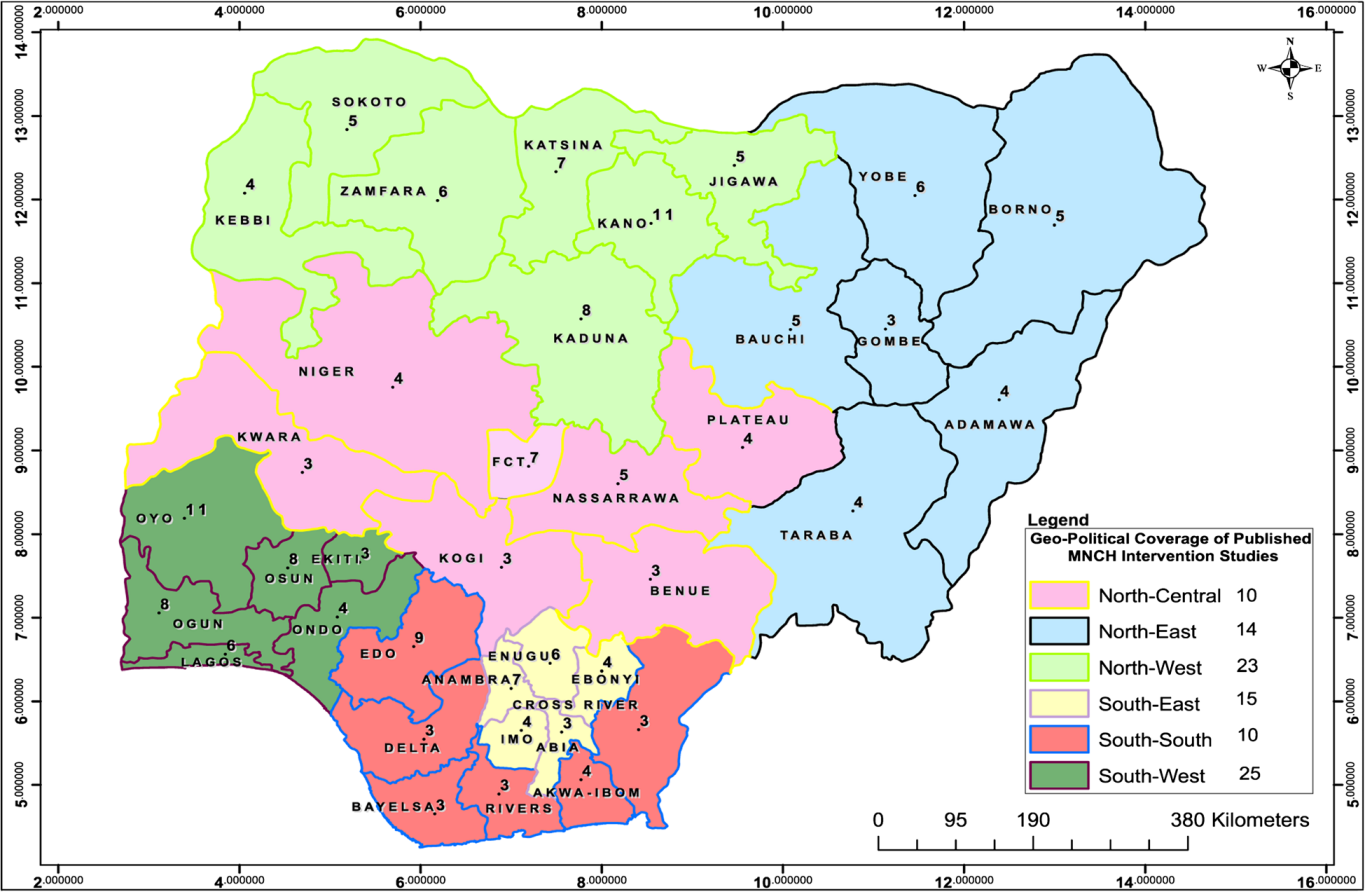
Map of Nigeria illustrating the geographical coverage of published MNCH intervention studies, 1990-2014.

The geographical distribution of the cumulative number of interventional studies per state in the six geo-political regions of Nigeria is displayed in Figure 5. South-western Nigeria is the geo-political region with the highest coverage (25) of published interventional studies. The north-eastern and the north-central sub-regions recorded 14 and 10 studies each whereas in the north-west, south-east and south-south the frequency of published studies for the period of observation was 23, 15 and 10 studies, respectively. Only three studies had national coverage whereas others were targeting two regions (6), three regions (3) and four regions (2).

### Characteristics and developmental trend of national policies, strategies and guidelines for MNCH

**Figure 6:**
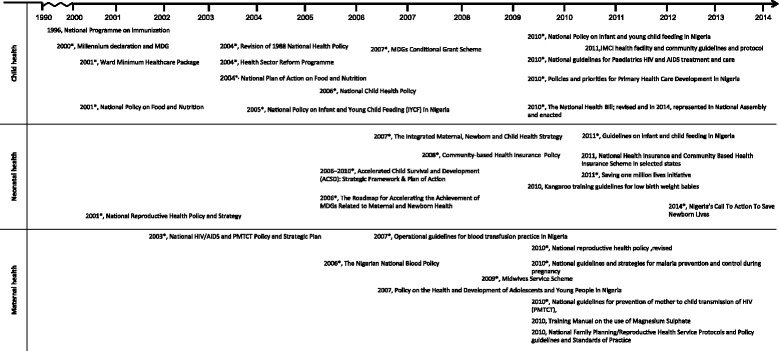
Chronology of the development of national MNCH policies, strategies and guidelines in Nigeria, 1988–2014.

Thirty-thirty policies and guidelines for MNCH were introduced in Nigeria since the development of the National Health Policy in 1988 with a revision done in 2005 (Figure 6). Although the intervening period (1990–1999) had little development, there has been a steady increase in the number of policies and revisions since 2000. The relationship between the intervention studies and development of policies is mixed. A clear example of evidence derived from local intervention studies preceding policy or guideline is the training manual on the use of Magnesium Sulphate (2010). There was multi-centric intervention studies conducted between 2006–2008 [60,62], which produced favourable results about the effectiveness of Magnesium Sulphate in the management of pre-eclampsia. On the other hand there are interventions [22], which were executed after the development of the integrated maternal, newborn and child health strategy (IMNCH; 2007). On the whole many of the policies have no temporal association with the intervention studies.

## Discussion

This systematic review presents a cross-sectional description and an indirect measurement of the coverage and effectiveness of the published MNCH interventions implemented in Nigeria since 1990. During the 24 years of observation, the Government of Nigeria, academic/health institutions, and development partners have jointly implemented and published a number of intervention projects to promote MNCH [18,20,22,30,31,49-108]. Results from this systematic review showed that majority (90%) of the interventions were commenced after 2000 and 65% were published between 2010 and 2014. This development may have been influenced by the increased awareness and commitment by the national Government and health authorities after the Millennium Declaration [109]. Our findings show that since 2000 there has been a simultaneous improvement in the development of MNCH policies, guidelines and strategies with that of intervention implementation and publication. Another explanation is the coincidence in the progressive increase of foreign aid and technical support to Nigeria since 1999 when there was restoration of democratic governance [110].

### Measurement of coverage and effectiveness of interventions

In this study, we found that the national MMR has consistently reduced during the period of observation (1990–2014) with a marked decrease in the last decade that featured the preponderance of published studies (2004–2014). Although progress has been recorded since the 1990s in reducing maternal deaths, the number of women who die in pregnancy or from complications associated with childbirth remains significantly high in Nigeria [2]. In 2010, Nigeria is estimated to account for 14% of maternal deaths worldwide [1]. The maternal mortality trend in Nigeria is inconsistent with the global MMR reduction from 422 (358–505) in 1980 to 320 (272–388) in 1990, and was 251 (221–289) per 100,000 live births in 2008. The annual rate of decline of the global MMR since 1990 was 1.3% (1.0-1.5), which is at variance to our estimate for Nigeria (APC = −3.10%, 95% CI: −5.20 to −1.00%), which was initially lower but an improvement was observed in the 2000s that overlaps with the proliferation of MNCH interventions. [2].

It is advocated that the concentration and integration of maternal health interventions targeting high risk and vulnerable points during maternity care minimizes morbidity and mortality [111]. Our results show that 21 studies specifically reported interventions targeting pregnancy and childbirth, which are sensitive periods for preventing maternal mortality [112]. In terms of outcome of interventions, our study recorded that maternal health promotion and prevention of complications interventions were targeted by 12 and 21 studies respectively. Furthermore, only 13 studies that reported interventions targeting health system strengthening, which has the potential of wider coverage and sustainability [113].

Nigeria is comprised of complex geographic and demographic characteristics as seen in the significant regional variation in maternal health indicators [16]. The extremely poor north-east region has an estimated MMR of 1,549 deaths per 100,000 live births, more than five times the global average [16]. Our findings showed that only three regional specific and 10 national or multi-regional published studies of MNCH interventions were recorded in the north-east. The recent terrorist attacks in the north have led to high levels of insecurity thereby creating an unfavorable environment for implementing health intervention programmes. Development partners have in many cases shut down or scaled back operations in the north and public health experts fear that any prolonged insecurity will attenuate the health gains of the last decade [32].

As for child health interventions, only 18 studies specifically targeting under-five children were published during the 24 year-period of observation. The downward trend in the under-five mortality also corresponds with the implementation and publication of most of the MNCH intervention studies in Nigeria. The regional variation in under-five mortality is accentuated by the consistently higher rates in the northeast and the northwest sub-regions. Our findings showed that these regions did not experience significant reduction compared to the other regions. The findings of the present study show that the included studies targeting under-five children were child health promotion and prevention of childhood diseases were few. The findings of this study also showed that the proliferation of policies and strategies targeting child health were mostly between 2005–2010. It is known that it requires time and other resources for health policies to be translated into change [114]. Our study has showed that 38 (67.3%) MNCH interventions were implemented for less than two years. The limited improvement in child health may be partly explained by the late take off, lack of sustenance and disjointed design and non-scaling up of implementation of interventions targeting MNCH.

Currently, policy recommendation favours MNCH interventions be designed and implemented to address fundamental etiological factors of the mother and child through a comprehensive and continuum of care approach [111]. Efforts to adopt this paradigm so as to improve the health status of mothers and children have been made by the Nigeria Federal Government in collaboration with national and international donors and partners. In 2007, the National Integrated Maternal, Newborn and Child Health (NIMNCH) Strategy was adopted.

Maternal, newborn, and child health outcomes are determined by multiple factors that require multi-pronged interventions operating synergistically to reinforce an overall positive effect [111]. Additionally, it is important to consider MNCH intervention outcomes and impact in terms of coverage, cost-effectiveness and benefit at the population level. It is reasonable that in a resource constrained setting, interventions should be designed to ensure efficiency and cost-effectiveness. Our results showed that only two studies reported on economic outcome (e.g. cost analysis) of interventions. In addition, it is challenging to demonstrate effectiveness of most interventions since only 35% of all the published intervention studies had a control or comparison group. This finding emphasizes the role of implementation/operational research and intervention trials/pilots before scale-up especially for the design and implementation of the post-2015 MNCH intervention projects.

### Study limitations

The indirect measurement of coverage and effectiveness described in this paper is limited to only studies that were published and available on the major search engines employed for this systematic review. Although many interventions have been implemented in Nigeria since 1990, attempts to get an inventory of all implemented interventions were unsuccessful. Therefore, some of these interventions may not have been included in this systematic review because they were unavailable for selection or did not meet the inclusion criteria. The barriers to the dissemination and timely application of research findings in the making of decisions about health care are complex and have been little studied [115]. Government and Funding agencies require the evaluation and documentation of implementation results only as programmatic processes [116]. Consequently, these evaluation reports don’t get published in academic journals because professional researchers who are motivated to publish did not execute them. This view is supported by our findings that showed 63% of the published studies having authors affiliated with Nigerian academic/health institutions and foreign donors/implementing organizations.

## Conclusion

This systematic review has provided important lessons for operational research and the application of epidemiological reasoning to the understanding of MNCH problems and institution of relevant interventions. Firstly, the prolonged intervention-publication interval may contribute to delayed management awareness, mobilization of resources and response. We also observed a lack of coordination of policies and interventions either as source of evidence for initiating intervention or its evaluation. Furthermore, the scale and duration of many of the interventions was insufficient to have demonstrable impact on maternal and child health outcomes. A number of the MNCH interventions were implemented as pilots or within the framework of vertical programmes thereby raising concerns for scaling-up for wider coverage, integration into the health system and sustainability.

Despite the limitations associated with the systematic review, the methodology employed ensured that the search strategy covered several sources. The selected studies may not be representative of all implemented interventions but they have revealed interesting findings about the coverage and effectiveness of MNCH interventions since 1990. Future systematic reviews should focus on grey literature and other data sources in order to improve the sample size and variety of included studies. This systematic review has also shown that more MNCH intervention research and publications of findings is required to generate local and relevant evidence. Policy and implementation will benefit from this development.

## Additional files

Additional file 1:
**Completed PRISMA checklist for reporting systematic reviews.**
Additional file 2: Table S1. Data extraction tool and included studies’ characteristics. Geographical location is classified as NC, NE, NW, SE, SS and SW or State and Local Government Areas (LGA). *NC = North central, NE = North east, NW = North west, SE = South east, SS = South south and SW = South west (regions of the country). ******CYP = couple years of protection***EmOC = Emergency obstetrics care****YLS = Years of life saved *********MIP = Malaria in p ******CORP = Community oriented resource persons ***********NASG = Non Pneumat. [18,20,22,27,30,31,49-60,62-71,73-108].
