# Supplementary material for: Maternal and child health interventions in Nigeria: a systematic review of published studies from 1990 to 2014
Source: BMC Public Health. 2015 Apr 9;15:334. doi: 10.1186/s12889-015-1688-3 (PMC4429684; doi:10.1186/s12889-015-1688-3)
Supplement: Additional file 2: Table S1. — Data extraction tool and included studies’ characteristics. Geographical location is classified as NC, NE, NW, SE, SS and SW or State and Local Government Areas (LGA). *NC = North central, NE = North east, NW = North west, SE = South east, SS = South south and SW = South west (regions of the country). **CYP = couple years of protection***EmOC = Emergency obstetrics care****YLS = Years of life saved *****MIP = Malaria in p ******CORP = Community oriented resource persons *******NASG = Non Pneumat. [18,20,22,27,30,31,49-60,62-71,73-108]. [file 12889_2015_1688_MOESM2_ESM.doc]

**Additional file 2: Table S1** Data extraction tool and included studies’ characteristics

| Author reference  and publication Year | Geographical location* and setting of implementation | Study design | Population | | Intervention | | Implementing organizations and sponsor | Year and duration of implementation | Outcome |
| --- | --- | --- | --- | --- | --- | --- | --- | --- | --- |
|  |  |  | Study | Selection criteria  and sample size | Intervention Group | Control/Comparison |  |  |  |
| Interventions targeting Adolescents & Pre-Pregnancy | | | | | | | | | |
| Abdul-Hadi, 2013 [52] | NE; Gombe; 2 unspecified rural LGAs | Pre/post quasi-experimental/ Community based intervention | Women of child bearing age in the study area | Unspecified | Community based distribution (CBD)of injectable contraceptives using community health extension workers | Nil | Unspecified foreign donor | 2009-end point unspecified Six months | The CBD mean CYP** for injectables- depomedroxy-progesterone acetate (DMPA) and norethisterone enantate was higher (27.72 & 18.16 respectively) than the facility CYP (7.21 & 5.08 respectively) (p < 0.05) with no injection related complications. The CBD's mean CYP for all methods was also found to be four times higher (11.65) than that generated in health facilities (2.86) (p < 0.05). |
| Speizer, 2014 [77] | NC, NW, SS and SW; Kaduna, Abuja-FCT, Kwara, Oyo and Edo states; Kaduna, Zaria, Ilorin, Abuja, Ibadan and Benin-City | Longitudinal evaluation | Women of child bearing age in  the study area | 4,303 women were interviewed at both baseline and  midterm, regardless of their marital status at either time period | Demand creation activities: 1. Social mobilization through interpersonal communication activities 2. Mass media with a particular focus on local and state-level radio programs that promote local-language family planning slogans 3. Program brand/slogans/logos were used across program activities | Nil | Urban RH Initiative funded by Bill & Melinda Foundation, supported by Measurement, Learning & Evaluation (MLE) project, University of North Carolina at Chapel Hill Carolina Population Center in collaboration with the International Center for Research on Women. | 2009; 5 years | After two years post-intervention outreach by community health or family planning workers as well as local radio programs were significantly associated with increased use of modern contraceptive methods. Television programs had a significant effect on modern contraceptive use. |
| Aja, 2011 [59] | SE, State, LGA  and setting unspecified | Pre/post quasi-experimental/ Community based intervention | Leaders of Christian women group belonging to the selected churches | 30 participants representing 15 Christian women’s groups including Anglican, Baptist, Presbyterian, Assemblies of God, Catholic,  Seventh-Day Adventists and other ministries and fellowships | Six groups of five participants each used the Women and Health Learning Package (WHLP) to create and develop a dialogue on adolescent health, a drama on violence against women, a song on nutrition and women’s health, a story on use of medicines by women, a quiz on cervical cancer and a poster on family planning groups | Nil | Global Health Through Education, Training and Service (GHETS) | Year unspecified 3 months | 13 of the 15 churches submitted a written report of the workshop to their local churches 1 month after the workshop as well as a copy to the workshop facilitator. Of the 13 churches, 3 organized a workshop to increase awareness on women’s health issues in their local churches within 3 months of the workshop |
| Okanlawon, 2011 [85] | SW; Oyo; Saki; Rural | Pre - and post – intervention quasi - experimental | Adolescents in  of the secondary school in the study area | Selected adolescents | Peer education programme for six months | Pre and post differential data in the experimental and control groups were compared | University of Ibadan, Ibadan, Nigeria | 2011; 6 months | The intervention had significant effect on adolescents in the experimental group compared with the control group in the area of knowledge of reproductive health issues (F1, 519) = 37.117, (p < .05). |
| Erim, 2012 [27] | NE and SW | Post-intervention evaluation of benefits  and cost-effectiveness using the  computer-based model; the Global Maternal Health Policy Model | All women of child bearing residing in the study area during the study period | Cohort of women  of child bearing age in 2006-2007 | Individual and integrated packages of interventions to prevent pregnancy-related deaths. Country- and region-specific data derived from 2008 NDHS were used for the economic outcome evaluation | Nil | Government of Nigeria | 2006-2008;  1 year | Increasing family planning was the most effective individual intervention to reduce pregnancy-related mortality, was cost saving in the Southwest zone and cost-effective elsewhere, and prevented nearly 1 in 5 abortion-related deaths. However, with a singular focus on family planning and safe abortion, mortality reduction would plateau below MDG 5. Strategies that could prevent 4 out of 5 maternal deaths included an integrated and stepwise approach that includes increased skilled deliveries, facility births, access to antenatal/postpartum care, improved recognition of referral need, transport, and availability quality of EmOC*** in addition to family planning and safe abortion. The economic benefits of these strategies ranged from being cost-saving to having incremental cost-effectiveness ratios less than $500 per YLS****, well below Nigeria’s per capita GDP |
| Hotchkiss, 2011 [88] | Whole country; urban and rural areas | Evaluation of expansion of the role of private providers in the  provision of modern contraceptive supplies and its association with increased horizontal inequity in modern contraceptive use. The study is based on multiple rounds of Demographic and Health Survey data | Women of reproductive  age (15 to 49 years of age) | The final sample consists of women  of reproductive age who are either currently married or living in union | Expansion of the private commercial sector in the provision of contraceptive supplies | Nil | Government of Nigeria and unspecified development/ private organizations | 1999-2008; 10 years | The percent of women who report currently using modern contraceptive methods declined, from 8.6 percent in 1999 to 8.1 percent in 2008, while the private commercial sector share increased from 34.6 percent to 58.4 percent over the same period. As the private sector’s provision of contraceptives increased from 1999 to 2008, MCPR inequity, as measured by the need-standardized distribution, decreased slightly from 1999 to 2003 (CI = 0.24 in 2003), and then increased from 2003 to 2008 (CI = 0.36 in 2008). |
| Gage, 2011 [90] | NC, NE, NW and SW; Bauchi, Federal Capital Territory (FCT), Kano, Lagos, and Nasarawa with 51 project LGAs;  urban and rural areas | Pre/Post intervention comparison of the 2005 baseline and the 2009 endline facility survey  to assess the degree of change in selected health indicators | Users of health service in intervention facilities | The target sample  size was 4,080. Allowing for 10 % non-response, the sample size was increased to 4,500. A multistage stratified sampling strategy was used for the household survey | Integration of the health  and education sectors through the promotion of community coalitions | Nil | Community  Participation for Action in the Social Sector (COMPASS) Project funded by U.S. Agency for International Development (USAID) | 2005-2009; 4 years | The percentage of health facilities with at least two modern contraceptive methods available at the time of the survey, a trained provider who had undergone at least basic family planning training to administer each and completed family planning records did not change significantly over time (15 % in 2005 versus 17 % in 2009) The percentage of health facilities that sold or distributed insecticide-treated nets (ITNs) declined significantly in Bauchi from 47 % in 2005 to 21 % in 2009 but increased significantly in FCT from 21 % in 2005 to 50 % in 2009. There was a significant increase over time in the availability of pre-packaged IPT in the total sample (58 % in 2005 versus 71 % in 2009) and in the KLN states (58 % in 2005 versus 69 % in 2009). The percentage of health facilities offering all six essential antigens for child immunization and possessing completed immunization records did not change between the baseline and end-of-project surveys. However, vaccination outreach by health facilities increased significantly during the inter-survey period, from 14 % in 2005 to 42 % in 2009. |
| Fayemi, 2011 [91] | 10 rural local government areas (LGAs) in five unspecified Nigerian states | Baseline and endline evaluation of  intervention | Women of child bearing age residing in the study area | Women who were reached by the intervention services | 250 community members were trained as community based distribution agents (CBDA) to provide information on reproductive health, provide non-prescriptive family planning (FP) commodities, treat minor aliment and make referrals to primary health centres within the communities | Nil | Government of Nigeria and unspecified donor | 3 years | Increase in the proportion of community members who had utilized FP commodities at all, from 28 % at baseline to 49 %, and an increase in the proportion of current contraceptive users from 16 % at baseline to 37 %. An average of 50 % increase in clientele patronage was also observed in the 10 LGAs’ primary health care centres. Most (96 %) of the interviewed CBDA agents reported that a drug-revolving system was in place to ensure that drugs and commodities were available. On-the-spot assessment of the service forms revealed that 86 % of them had their activities regularly recorded in their worksheets. Some of the challenges faced by CBDA were discrimination and misconception of community members about family planning (38 %), inadequate financial support (14 %),and transportation problems (8 % |
| Asekun-Olarinmoye, 2008 [95] | SW; Osun; Shao; Rural | Pre/Post-Intervention (quasi-experimental) study | Adult males and females in the study area | Multistage sampling technique | Health education | Nil | Ladoke Akintola University of Technology | Unspecified | Most respondents (88.0 %) cited traditional excisors as operators of the procedure, while 7.8 % mentioned health workers. Factors found to be statistically significantly associated with the practice of FGM are age, gender and educational status of respondents (p < 0.05). The age at which FGM is usually performed was put at under one year old by 60.3 % of respondents. All respondents cited type II FGM as the type practiced in the community. Most (88.0 %) of the female respondents were excised. A greater proportion of men than women did not want the practice of FGM stopped in the pre-intervention stage; however, there was a statistically significant decrease in the proportion of males who did not want the practice of FGM stopped in the post-intervention stage. Also, there was a statistically significant increase in the proportion of respondents who had no intention to excise future female children in the post-intervention stage (p < 0.05). Legislation, female literacy and empowerment, educating men and provision of alternative vocation for excisors were means suggested by respondents for stopping the practice |
| Mba, 2007 [99] | SE; Rural | Pre/Post Intervention study | Adolescents resident in the study  community | A total of 180  students selected by systematic sampling from each of the two randomly selected schools | Impact of reproductive health education on the knowledge and attitude of adolescents | Another secondary school (control group), which did not receive any intervention | The Communication Initiative Network and Partnership | 6 weeks | All the respondents have heard of reproductive health and could identify at least one of its components, their knowledge of it prior to the health education were defective and were obtained mainly from peers and the mass media. Such information was incomplete and often coloured with cultural and religious bias. However, there was a significant (p < 0.05) gain in correct knowledge following the health education. The students in the study group showed a positive and permissive attitude towards reproductive health education and there was a drop in risky sexual behaviour following the intervention. Pre-marital sex (94.3 %), pregnancy prevention and abortion (88.5 %) and sexually transmitted infections (82.8 %) were common reproductive health problems raised by the students. |
| Adesina, 1999 [100] | SW; Osun | Post intervention impact evaluation | Women of childbearing  ages and their husbands resident in the study area | Selected women of childbearing ages  and their husbands | Presentation of seminars  and lectures in churches, town halls, and guild houses on all aspects of EOC; presentation of a video film regarding problems on EOC; and organization of clinics to further educate women of childbearing ages and their husbands | Nil | Center for Research, Evaluation Resources, and Development | 8 months | Intervention materials were effective and have been well circulated in the communities. However, a longer period of intervention is needed in order to change established practices. Furthermore, the study suggests that the Nigerian government needs to put in place some policy guidelines that would help change the attitudes of men to their pregnant wives. One specific example is a policy that encourages men to attend clinics just like their pregnant wives |
| Ministry of Health, 1993 [105] | SW; Oyo; Rural | Impact evaluation | Women of child bearing age in intervention area | Trained CBD  workers and operating in intervention areas | Community-based distribution (CBD) of low cost family planning and maternal and child health services in rural | Nil | Government of Nigeria, Pathfinder Fund, University College Hospital Ibadan, and the Center for Population and Family Health of Columbia University | 1980-1985;  5 years | Initial family planning (FP) acceptance was low; ever use of a modern method has increased from 2 to 25 % in the pilot area. About half of the married women of reproductive ages in the project area are not sexually active at any one time because of postpartum abstinence. Most of the acceptance of modern contraceptives replaces use of traditional abstinence. Male promoters have proved to be an asset to male acceptance of FP services. Individual monetary incentives are not required to motivate CBD workers; however, once incentives are given, difficulties are created if they are stopped, as they were in the pilot area. The CBD approach has changed the concept of health care from that of providing services to clients who come to a fixed site to reaching out to provide services to all people living within a particular catchment area |
| Interventions targeting pregnancy | | | | | | | | | |
| Ogu, 2012[54] | 8 states in NC, NE & NW, Niger, Kaduna, Kano, Adamawa, Bauchi, Borno, Taraba, and Katsina; rural and urban LGAs | Pre/Post-intervention quasi-experimental/ Hospital based intervention | All women of child bearing  age residing in project areas | 8 project states that were selected  because of their  poor maternal health indicators compared to national average | Capacity-building workshops consisting of 5-day training sessions for private medical practitioners to improve the quality of private sector provision of post-abortion care | Nil | Women’s Health and Action Research Centre (WHARC), Lucile and David Packard Foundation, Pathfinder with funding from the Ford Foundation | 2002-2012 10 years | 458 trained providers in 430 private clinics treated a total of 17,009 women over the 10 years of the project (about 2,100 women annually). Not a single case of abortion-related maternal mortality was recorded |
| Mens, 2011 [70] | SS, Edo Owan-East and Akoko-Edo, Rural | Pre/Post-intervention quasi-experimental/Community based intervention with control arm | Women of  child bearing  age | Within each of the LGAs three clusters/ communities were selected based on urban status and geographical  position | A peer education  campaign was developed  in order to increase level  of knowledge about  adverse health effects of malaria during pregnancy and uptake of preventive practice among women of child bearing age | Baseline and post-intervention  assessment of knowledge and prevention practices during pregnancy | University of Benin Teaching Hospital | 2009 6 months | In the pre-assessment women on average answered 64.8 % of the question on malaria and its possibility to prevent malaria correctly. The peer education campaign had a significant impact in raising the level of knowledge among the women; after the campaign the respondents answered on average 73.8 % of the questions correctly. Stratified analysis on pre and post assessment scores for malaria in general (68.8 & 72.9 %) and MIP***** (61.7 & 76.3 %) showed also significant increase. Uptake of bed nets was reported to be low: 11.6 % |
| McNabb, 2014 [78] | NC; Abuja-FCT  and Nasawara; Setting unspecified | Pre / post intervention study | Pregnant women attending ANC  in 10 selected study Primary Health Centres | 266 clients over 18 years were interviewed directly after their first ANC visit | The ten PHCs were equipped with phones or tablets and 150 CHEWs were trained to use the installed mobile application. The app dynamically guides health workers through ANC protocols and collects client data in real time. | Nil | Pathfinder International | 2012; I year | A quality score consisting of 25 indicators covering technical and counseling elements of ANC was developed. Client exit interview data was analyzed to assess change in overall quality score, as well as change in the provision of key elements of ANC. Quality score increased from 13.3 at baseline to 17.2 at the endline (p < 0.0001), out of a total possible score of 25, with the most significant improvements related to health counseling. In terms of technical elements, the quality score increased from 7.77 at baseline to 8.44 (p < 0.0001). The health education domain of the quality score had the highest increase from 5.45 to 8.67 points (p < 0.001). |
| Anyaehie, 2011 [31] | SE, Imo, Nkwerre, Rural | Pre/post-intervention quasi-experimental/ Community based intervention | Apparently healthy women attending antenatal, postnatal and immunization clinics in all the health centers located in the study area | Eligible women  were randomly recruited  by means of a lucky dip of yes or no. Subjects who have symptoms indicating malaria, for e.g. fever and headache, weakness, anorexia and joint/muscle pains were excluded from the study; 990 pregnant and nursing mothers, aged 18 – 39 years | Free ITN distribution  during antenatal, postnatal and immunization clinics | Nil | Government of Nigeria | 2007 1 year | There was a sustained but insignificant rise in asymptomatic malaria parasitemia post-distribution of ITNs. Out of the 990 subjects recruited, 470 tested positive with the prevalence of asymptomatic malaria parasitemia of 47.5 %. Out of the 520 who tested negative for malaria parasite, 515 reported in the 2nd month, 501 in the 3rd month and 490 in the 6th month. |
| Chabikuli, 2009 [64] | All the states of Nigeria; Urban  and rural | Pre/Post-intervention quasi-experimental/Hospital based intervention | Women of child bearing age attending family planning, HCT, ART and PMTCT clinics in 71 public health facilities | 115 comprehensive ART sites and over 300 feeder clinics | Integrating family  planning with HIV counseling and testing (HCT), antiretroviral  therapy (ART) and prevention of mother-to-child transmission (PMTCT) in the Nigerian public health facilities | Nil | GHAIN and U.S. President’s Emergency Plan for AIDS Relief (PEPFAR) | 2007-2009 18 months | Mean attendance at family planning clinics increased significantly from 67.6 % in pre-integration to 87.0 % in post-integration. The mean CYP increased significantly from 32.3 pre-integration to 38.2 post-integration. Service ratio of referrals from each of the HIV clinics was low but increased in the post-integration period by 4, 34 and 42 per 1000 clients from HCT, ART and PMTCT clinics, respectively. Service ratios were higher in primary healthcare settings than in secondary or tertiary hospitals. Attendance by men at family planning clinics was significantly higher among clients referred from HIV clinics. |
| Kalu, 2012 [82] | SE; Ebonyi; Abakiliki; urban | Hospital-based post-intervention evaluation | Post-Abortion Care (PAC) service  providers in  study facility | A standardized questionnaire was administered to 45 direct PAC service providers | Provision of post-abortion care and effective linkage to other post abortion services | Nil | Ebonyi State University Teaching Hospital | 2004-2009;  5 years | Abortion complications constituted 41.4 % of all Gynaecological admissions. Maternal mortality from complications of abortion was 11.5 % of all the maternal mortality at the centre. Women aged 19 years and less were 37 (7.1 %) and single women were 132, constituting 25.3 % of all cases. About 31 % of the PAC care providers had formal training for the implementation of the PAC services. Fifteen percent of the caregivers were satisfied with the linkage between PAC and the Family Planning services. There is poor integration between emergency post abortion care and other reproductive health services in the centre |
| Joseph, 2014 [86] | SS; Edo;  Benin-city; Urban | A cohort study | 249 HIV  infected  women who  had intrapartum care | Women who  received HAART early in pregnancy | Administration of highly active antiretroviral  therapy (HAART) from early pregnancy | Unbooked HIV  positive pregnant women, who had not received antiretroviral drugs during the antenatal period but received nevirapine in labour, referred to as untreated-maternal HIV infection | University of Benin Teaching Hospital | 2008-2009;  1.5 years | Intrauterine growth restriction (IUGR) (20.5 % vs. 6.3 %, p = 0.003), pre-term birth (25.0 % vs. 9.8 %, p = 0.005) and caesarean delivery (45.5 % vs. 29.8 %, p = 0.04) were significantly higher among women with untreated-HIV infection in pregnancy compared with women who received HAART from early pregnancy. Untreated maternal HIV-infection was associated with higher frequency of birth weight less than 2500 g, 5-minutes Apgar score less than 7 and admission into neonatal unit (p < 0.05). Women with primary education were significantly higher in the group with untreated maternal HIV infection (27.3 % vs. 12.7 %, p = 0.003) |
| Chiwuzie, 1997 [103] | SS; Edo; Ekpoma | Intervention process  and output evaluation | Clan heads, women of child bearing age and health workers in study area | Community members managing emergency funds, women with complications that have accessed the funds and health workers who have attended to them | Emergency loan funds to improve access to obstetric care | Nil | University of Benin, Benin City, Nigeria | 1995 | Of the 13 clans contacted, 12 successfully launched loan funds. Total donations amounted to US$793, of which the community contributed four-fifths. In the 1st year of the operation, 456 women/families requested loans (ranging from US$7 to US$15), and 380 (83 %) were granted. Three hundred and fifty-four (93 %) loans were repaid in full. In addition to being used for transport, loans were used to help pay for drugs, blood and hospital fees. Costs: The cost of establishing the loan fund was US$1360, including initial donations to the loan funds. The PMM project paid 55 % of the total |
| Okeibunor 2011 [75] | SS, Akwa Ibom, Eket, Esit Ekit, Onna LGAs (Interventional) and Ikot Abasi, Mbo, Mkpat Enin LGAs (Control); Rural | Before and After  parallel group design | All pregnant women residing  in programme areas | Women who had given birth within 6 months. 1,280  women aged  15–49 with recent pregnancies in the study areas were randomly selected  for an interview at baseline. A second, independent sample  of 1,380 women was randomly selected post-intervention | 3 groups received community directed intervention (CDI) for delivery of ITNs and two doses of SP for IPTp and basic counseling services  for pregnant women by community directed distributors (CDD) Non-random group assignments at local government level by for balanced samples. | 3 groups had no CDI. Both Intervention  group and control had increased support for public health facilities (training, resources, supplies) | Government of Nigeria, Johns Hopkins University, with funding support from ExxonMobil Foundation | 2008, 2 years | Relative to women in the control area, an additional 7.4 percent of women slept under a net during pregnancy in the treatment areas (95 % CI [0.035, 0.115], p-value < 0.01), and an additional 8.5 percent of women slept under an ITN after delivery and prior to the interview (95 % CI [0.045, 0.122], p-value < 0.001). The effects of the CDI programme were largest for IPTp adherence, increasing the fraction of pregnant women taking at least two SP doses during pregnancy by 35.3 percentage points [95 % CI: 0.280, 0.425], p-value < 0.001) relative to the control group. No effects on antenatal care attendance were found |
| Interventions targeting child birth | | | | | | | | | |
| Tukur, 2012 [18] | NW, Kano, 10 General Hospitals located at Kano, Bichi, Wudil, Gwarzo, Rano, Minjibir, Tudun Wada, Doguwa, Rano, and Rogo LGAs. Apart from Kano, the rest were rural towns | Pre-post-intervention quasi-Experimental/ Hospital based intervention | All pregnant women residing  in intervention areas | Hospitals were selected on the basis of geographic spread across the state, population, and high burden of maternal deaths | 1,045 health workers (Doctors and midwives) were trained on introduction of magnesium sulphate (MgSO4) for preeclampsia and eclampsia | Nil | Population Council  with funding from MacArthur Foundation | 2008 1 year | In 1 year of intervention, a total of 1,045 patients with severe preeclampsia and eclampsia were treated. The case fatality rate for severe preeclampsia and eclampsia fell from 20.9 % (95 % CI 18.7–23.2) at baseline to 2.3 % (95 % CI 1.5–3.5) post intervention. The perinatal mortality rate was 12.3 % as compared to 35.3 % in a center using diazepam |
| Prata, 2012 [50] | NW, Kaduna,  Zaria, peri urban | Pre/Post-intervention quasi-experimental/ Community based intervention | All pregnant women residing  in intervention areas | 5 communities near Zaria in the northwestern  Nigeria. The study communities are all situated in the northern section of Kaduna State. Total estimated population was approximately 21,000, with most residing in the peri-urban slum of Hayin Dogo (8,940 residents), and the fewest in Tsibiri (1,490). Eventually 1,800 postpartum women interviewed | Community mobilization and health education about birth preparedness and the prevention of postpartum hemorrhage through prophylactic use of misoprostol | Nil | Unspecified foreign donor | 2009 1 year | A total of 1,875 women were enrolled in the study in 2009. Most women delivered at home (95 %) and skilled attendance at delivery was low (7 %). Community mobilization efforts reached most women with information about postpartum hemorrhage and misoprostol (88 %), resulting in high comprehension of intervention messages. Women identified TBAs and CORPs****** as the single most important source of information about misoprostol 41 % and 31 % of the time, respectively. Availability of misoprostol at the community level gave 79 % of enrolled women some protection against postpartum hemorrhage, which they otherwise would not have had. |
| Ojengbede, 2010 [62] | NW, SW, Kano, Katsina, Oyo,  Urban | Non-randomized  pre/post intervention | Pregnant women resident in the study area at the time of study | Selected women  with PPH due to uterine atony,  retained placenta, ruptured uterus, vaginal or cervical lacerations or placenta accreta with estimated blood loss of 6,750 ml and one clinical sign of shock. 288 women were evaluated in four referral facilities | Provision of  non-pneumatic anti-shock garment to women with postpartum hemorrhage | Nil | Government of Nigeria and unspecified foreign donor | 2004-2008 4 years | Mean measured blood loss decreased by 80 % between phases. Women experienced 350 ml of median blood loss after study entry in the pre-intervention and 50 ml in the NASG*******phase (p < 0.0001). Mortality decreased from 18 % pre- intervention to 6 % in the NASG phase (RR = 0.31, 95 % CI 0.15– 0.64, p = 0.0007). In a multiple logistic regression model, the NASG was associated with reduced mortality (odds ratio 0.30; 95 % CI 0.13–0.68, p = 0.004) |
| Hunyinbo, 2008 [67] | SW; Ogun; Abeokuta; Urban | Pre/post intervention quasi-experimental/Hospital based intervention | All patients with specific obstetric complications within the period of study | Inclusion criteria  were based on the working definition  of life threatening obstetric complications that include obstetric heamorrhage, eclampsia,  obstructed, labour, uterine rupture and genital tract sepsis. All patients with specific obstetric complications within the period of study were selected; 65 women were evaluated | Criteria–based clinical  audit in measuring and improving quality of obstetric care for five life-threatening obstetric complications: obstetric heamorrhage, eclampsia, genital tract infections, obstructed labor and uterine rupture | Nil | Federal Medical  Centre, Abeokuta, Nigeria | 2002-end  point unspecified 13 months | Following Phase I, areas in need of improvement were identified; mechanisms for improving quality of care were identified and implemented. Overall care of the complications improved significantly in obstetric heamorrhage (61 to 81 %, p = 0.000), eclampsia (54.3 to 90 %, p = 0.00), obstructed labour (81.7 to 93.5 %, p < 0.001) and genital tract sepsis (66 to 85.2 %, p < 0.01). Clinical monitoring, drug use, and urgent attention by senior medial staff also improved significantly after intervention. Criteria-based clinical audit is feasible and acceptable for improving management of life-threatening obstetric complications. |
| Ezugwu, 2014 [79] | SE; Enugu; Urban | Hospital-based impact evaluation | Pregnant women that gave birth at the study facility during the  time of study | The case files of all maternal deaths  were retrieved  (91 % retrieval rate) from the medical records department and relevant data including the age, parity, marital, booking, and educational status of women and the cause of deaths were extracted for analysis | Institution of evidence  based management guidelines for eclampsia  and post-partum hemorrhage | Nil | Enugu State University Teaching Hospital | 2005-2010; 6 years −3 years before (2005–2007) and after (2008–2010) intervention | There were 9150 live births and 59 maternal deaths during the study period, giving an MMR of 645/100 000 live births. Pregnant women who had no antenatal care had almost 10 times higher MMR. There was 43.5 % reduction in the MMR with the interventions (488 vs. 864/100 000 live births P = 0.039, odds ratio = 1.77). There was also significant reduction in case fatality rate for both eclampsia (15.8 % vs. 2.7 %; P = 0.024, odds ratio = 5.84 and Post partum hemorrhage (PPH) (13.6 % vs. 2.5 % P value = 0.023, odds ratio = 5.5. |
| Sutherland, 2013 [80] | NW, SW; Katsina, Oyo; Katsina, Ibadan; Urban | Pre/Post- intervention clinical trials of the  cost-effectiveness of a non-pneumatic  anti-shock garment (NASG) | Patients with obstetric hemorrhage of any etiology (ranging from ectopic pregnancy to ruptured uterus) were studied. | 1000 women presenting in shock. | Three intervention  scenarios were examined:  no women in shock  receive the NASG, only women in severe shock receive the NASG, and all women in shock receive the NASG | Comparison of costs  and disability-adjusted life years (DALYs)  were across the intervention scenarios | Government of Nigeria with funding from John D. and Catherine T. MacArthur Foundation | 2010; 1 year | Providing the NASG to those in severe shock results in decreased mortality and morbidity, which averts 2,063 DALYs. Differences in use of interventions result in net savings of $6,460, with a cost per DALY averted of $3.13. Results of providing the NASG for women in mild shock has smaller and uncertain effects due to few clinical events in this data set |
| Okonofua, 2013 [81] | Geographical location  unspecified | Pre/Post-intervention (Multi-center) intervention study | All pregnant women residing  in intervention areas | A total of 219 cases  of eclampsia were managed over a 12-month period | Doctors and midwives in  Six teaching hospitals  were re-trained to manage eclampsia using magnesium sulfate according to the Pritchard protocol | Nil | Government of Nigeria and unspecified donor | Year of implementation unspecified; 1 year | The post intervention case fatality rate of 3.2 % was significantly less than the pre-intervention rate of 15.1 % (p < 0.001). The overall maternal and perinatal mortality ratios and rates respectively in the hospitals declined from 1199.2 to 954 per 100,000 deliveries and 141.5 to 129.8 per 1000 births, respectively (p > 0.05) |
| Igwegbe, 2012 [83] | SE; Anambra; Nnewi | Hospital-based impact evaluation | All pregnant women that  gave birth at the facility during  the study period | Case notes of all maternal deaths recorded during the period of study | Implementation of the service Compact with all Nigerians (SERVICOM). Whose provisions included: quality services designed around the requirements of their customers and served by staff sensitive to the needs of their clients; commitment to the provision of services within realistic time frames; provision of officials or agencies to whom complaints about service failures could be addressed; and periodic published surveys to determine levels of customer satisfaction. | Nil | Nnamdi Azikiwe University Teaching Hospital | 2004-2010; 6 years | There were 4916 live births and 54 maternal deaths during the study period, giving an MMR of 1098 per 100 000 live births. Pre-eclampsia/eclampsia was the most common direct cause (25.0 %), followed by hemorrhage (18.8 %) and sepsis (8.3 %). Anemia (12.5 %) was the most common indirect cause. There was a progressive reduction in MMR and RR of maternal mortality, with a corresponding increase in live births. The presentation–intervention interval improved significantly from 2006. |
| Galadanci, 2011 [89] | NW; Kano and Kaduna | Process/ Post-intervention outcome evaluation. Continuous maternal and fetal data collection and analysis were conducted from 2008 to 2009 by means  of a maternity record book and structured monthly summary form | Pregnant women attending any od the 10 quality assurance project hospitals for improving maternal and fetal outcome | Women who gave birth in project hospital and their infants | Continuous monitoring of quality assurance in maternity units | Nil | Government of Nigeria and unspecified private donor | 2008-2009; 1 year | The mean maternal mortality ratio (MMR) was reduced from 1790 per 100000 births in the first half of 2008 to 940 per 100000 births in the second half of 2009. The average fetal mortality ratio (FMR) decreased slightly from 84.9 to 83.5 per 1000 births. There was an inversely proportional relationship between the total number of deliveries in a hospital and MMR and FMR. There was a close correlation between the MMR and the equipment status and hygiene conditions of the hospitals |
| Asa, 2008 [96] | SW; Osun; Ile-Ife; Enuwa; Rural | Randomized controlled trial | Pregnant women receiving antenatal care at the study facility | 352 primigravid and secondigravid  women between  16 and 30 weeks gestation | Efficacy of intermittent preventive treatment of malaria using  sulphadoxine-pyrimethamine (SP) | Treatment doses of  CQ at recruitment and subsequently only if they had symptoms suggestive of malaria | Obafemi Awolowo University, Ile-Ife, | 1 year | At recruitment and 34 weeks gestation, there was no statistically significant difference between the experimental and control group in terms of socio-demographic characteristics and past medical history. Thirty-three (22.6 %) and 52 (37.1 %) women in the study and control groups, respectively, had anaemia (protective efficacy 49.5 %, p = 0.01). With multivariate analysis, controlling for the possible confounding effects of education, parity, haemoglobin level at booking and malaria parasitaemia in peripheral blood, the difference in the incidence of anaemia in the two groups remained significant (p = 0.01; odds ratio = 0.5; 95 % confidence interval = 0.29-0.85) |
| Gummi, 1997 [106] | NW; Kebbi; Maiyama, Jega | Pre/Post-Intervention evaluation by  institution and community-based situation analysis studies using quantitative and qualitative methods | Women of child bearing age in study area | Targeted  communities and health facilities.  All the women who gave birth during  the period of intervention | Community education to encourage use of  emergency obstetric services.  The project interventions involved 1) material improvements in the health facilities, 2) staffing improvements, 3) community education activities, and 4) improving transport mechanisms. The community education activities involved creation of a safe motherhood committee composed of local leaders, development of key messages and educational materials, creation of a videotaped docu-drama of the maternal death of a 17-year-old, and development of a project logo | Nil | Sokoto Prevention of Maternal Mortality (PMM) team/ International PMM Network | 1991-1995; 4 years | The pre-intervention studies indicated that maternal mortality rates were high and that most women delivered at home, required the permission of their husbands to seek outside care, and had accurately low opinions of the quality of care in the health facilities. The intervention increased the community's awareness of the causes of maternal death, nature of obstructed labor, signs of pre-eclampsia, need for prompt treatment, and importance of delaying marriage. Utilization of the Maiyama Maternity Center also increased during the project. The number of women referred from the Center increased from 18 in 1992 to 35 in 1993 and then declined sharply. Utilization of the Jega Health Center increased from 1991 to 1993, declined in 1994, and increased somewhat in 1995. Referrals from this facility remained stable. Treatment rates at the hospital also fluctuated, but the case fatality rate declined from 38 % in 1991 to 5 % in 1995. The cost of the intervention was US$9535, with 96 % contributed by the project and 4 % by the community. It is concluded that the increased com |
| Miller, 2009 [60] | NW, Katsina, Katsina, Urban | Non-randomized pre-intervention/  intervention | Pregnant women resident in the study area at the time of study | Entry criteria were obstetric hemorrhage (>or = 750 mL) and a clinical sign of shock (systolic blood pressure <100 mm Hg or pulse >100 beats per minute). Women were enrolled in a pre-intervention phase (n = 83) and an intervention phase (n = 86) at a referral facility in Katsina, Nigeria. | Provision of non-pneumatic anti-shock garment to women with postpartum hemorrhage | Nil | Government of Nigeria and unspecified foreign donor | March 2004 - December  2007; 3 years  9 months | Mean measured blood loss in the intervention phase was 73.5+/−93.9 mL, compared with 340.4+/−248.2 mL pre-intervention (P < 0.001). Maternal mortality was lower in the intervention phase than in the pre-intervention phase (7 [8.1 %]) vs 21 [25.3 %]) (RR 0.32; 95 % CI, 0.14-0.72) |
| Interventions targeting Postnatal (mother and newborn) | | | | | | | | | |
| Ikechebelu, 2011 [87] | SE; Anambra; Nnewi | Prospective descriptive study (longitudinal evaluation) | Nursing mothers and their babies attending  PMTCT clinic  of the study site | Seven hundred and twenty six mother-infant pairs managed in the PMTCT programme of the study facility | The babies HIV status was tested with PCR for HIV DNA while the mothers provided information on infant feeding pattern and the use of antiretroviral (ARV) drugs including prophylaxis for the baby | Mothers who did not receive HAART,  did not breastfeed and whose babies did not received ARV prophylactic therapy. | Nnamdi Azikiwe University Teaching Hospital | 2011 | The transmission rate was 2.8 % for mothers, who were on HAART, did not breastfeed and whose babies received ARV prophylactic therapy. But for mothers who did not receive HAART, did breastfeed and whose babies did not received ARV prophylactic therapy, the transmission rate was 37.5 %. When both the mother and child received ARV drugs, the transmission rate was significantly lower in those who did not breastfeed (2.8 %) than in those who breastfed (12.5 %)(P < 0.001). When both the mother and child did not receive ARV drugs, the transmission rate significantly lower in those who did not breastfeed (21.1 %)than in those who breastfed (37.5 %) (P < 0.02) |
| Interventions targeting Infancy and childhood | | | | | | | | | |
| Gwarzo, 2012 [53] | NW, Kano, Gezawa,  rural LGA | Pre/Post-interventional quasi-experimental/ Community based intervention | Under-five children | The intervention site was selected based  on the analysis of data on the uptake  of polio vaccination across Nigeria was done to identify the state with the lowest uptake and highest number; 11,847 under-five children were targeted | Social mobilization and health education using video road side film show conducted in communities by mobile vans to promote Immunization uptake | Nil | Government of Nigeria | 2008 6 months | The baseline polio vaccination uptake among children under five, and the number with zero doses (never received polio vaccination in the past), from 4 settlements combined were 2,755 and 125, respectively. At 6-month post-intervention, the number vaccinated and zero doses detected were 11,364 and 88, respectively; producing a relative increase of about 310 % in the polio vaccination uptake and a net reduction of 29 % of never vaccinated children |
| Fatungase, 2012 [56] | SW;Ogun; Ijebu North | Pre/Post-interventional quasi-experimental/ Community based intervention with a control group | Caregivers of under-five children | Only mothers or guardians who were permanent residents (resident in the area >6 months) and currently having children under 5 living with them were included in the study | Health education training  on the home management  of Malaria for the caregivers of children  under 5 years old | Baseline and post-intervention  assessment of knowledge and practice of home management of malaria | Olabisi Onabanjo University, Sagamu, Nigeria | 2010 3 months | The majority in both the experimental (75.0 %) and control (71.5 %) groups use artemisinin-based combination therapy as first line home treatment drugs pre intervention. Post health education intervention, the degree of change in the knowledge of referral signs and symptoms in the experimental group was 52.8 % (p < 0.0001) while it was 0.2 % in the control group (p = 0.93). Tepid sponging improved by 45.0 %, paracetamol use by 55.3 %, and the use of herbs and other drugs were not significantly influenced in the experimental (p = 0.65 and 0.99) and control group (p = 0.89 and 0.88), respectively. Furthermore, there was a 55.7 % (p = 0.001) increase in the proportion of respondents using the correct dose of artemisinin-based combination therapy in the home management of malaria and 23.9 % (p < 0.001) in the proportion using it for the required time |
| Okeke, 2010 [65] | SE; Enugu; Iyiukwu; Rural | Pre/Post interventional quasi-experimental/ Community based intervention | Caregivers of under-five children in  study communities | The study communities are holoendemic for malaria and made  up of 10 villages  with a population of about 13,952; 30 leaders of women groups who subsequently trained other mothers in their group | Training programme for caregivers of under-five children to improve the recognition of malaria, treatment and referral | Nil | University of Nigeria Teaching Hospital, Enugu (UNTH) | 2003-end point unspecified 18 months | Post-intervention evaluation of the programme showed significant (p < 0.05) improvements in knowledge, home management of malaria and referral practices for severe malaria. Those who correctly reported that mosquitoes were the cause of malaria rose markedly from 39.7 % to 88.7 %. Knowledge of symptoms of mild and severe malaria also increased significantly. Only 1.5 % of caretakers were aware of the correct dose of anti-malarial before intervention, but this increased to 41.5 %. The impact of intervention brought about a dramatic change in the practice of taking severely ill children, especially those with convulsion, to a traditional healer. A minority (6.7 %) of caretakers took a severely ill child to a traditional healer as against 60 % pre-intervention. There was also a significant increase in use of formal health facilities for the treatment of severely ill children. |
| Okeke, 2009[66] | SE; Enugu; Iyiukwu; Rural | Pre/Post-interventional quasi-experimental/ community based intervention | Patent medicine vendors in study area | The study communities are holoendemic for malaria and made up of ten villages with a population of about 13,952; 16 drug vendors belonging to the PMV association were trained | Drug vendor-training programme to improve treatment and referral practices | Nil | University of Nigeria Teaching Hospital, Enugu (UNTH) | 2003-end point unspecified 8 months | The intervention achieved major improvements in drug selling and referral practices and knowledge. Exit interviews confirmed significant increases in appropriate anti-malarial drug dispensing, correct history questions asked and advice given. An improvement in malaria knowledge was established and 80 % compliance with referred cases was observed during the study period. |
| Qureshi, 2011 [57] | NW, Sokoto,  Kware and  Bodinga, Rural | Pre/Post-interventional quasi-experimental/ Community based intervention with a control group | Mothers of  infant children  in study area | In both study and control group using  a combination of  simple and  systematic sampling methods, a one in eight sample of 179 mother-child pairs were recruited from each community. In homes without breastfeeding mothers at the time of counting, the next house was chosen. Given the existence of polygamy in Sokoto, whenever a situation was encountered in which there were more than one mother child pairs in a single house, one pair was selected by simple random sampling using the balloting technique. | Training of female volunteers educate  mothers about  breastfeeding during  home visits | Baseline and post-intervention  assessment of knowledge and  practice of exclusive breastfeeding | Unspecified foreign donor | Unspecified | At baseline, intervention and control groups differed significantly regarding maternal occupation (P = 0.07), and age of the index child (P = 0.07). 42 % of infants in the intervention group were up to 6 months old and about 30 % of them were exclusively breastfed. Intention to EBF was significantly associated with maternal age (P = 0.01), education (P = 0.00) and women who were exclusively breastfeeding (P = 0.00). After counseling, all infants up to 6 months of age were exclusively breastfed. The proportion of mothers with intention to EBF increased significantly with maternal age (P = 0.00), occupation (P = 0.00) and women who were exclusively breastfeeding (P = 0.01). Post-intervention surveys showed that source of information and late initiation of breastfeeding was not significantly associated with intention to EBF. Mothers who reported practicing EBF for 6 months, were older (P = 0.00) multi-parous (P = 0.05) and more educated (P = 0.00) compared to those who did not practice EBF. |
| Ajayi, 2008 [76] | SW; Oyo;  Ona-Ara; Rural | Pre/Post-interventional quasi-experimental/ Community based intervention with  control group | Under-five children with febrile illness  and their caregivers | Children reported to have developed  fever in the 24  hours preceding research assistant’s visit to the selected communities in the two arms of the study were enrolled consecutively over a period of nine months provided their parent/guardian gave verbal informed consent. In households with more than 1 child currently sick with febrile illness, each of them was enrolled as long as they met the inclusion criteria. In the 9-month period 88 and 74 mother/children pairs were enrolled in the control and intervention arms respectively. | Training on malaria treatment guideline on mothers’ adherence to correct treatment of malaria in children | Assessment of malaria parasitaemia | University of Ibadan /University College Hospital, Ibadan | 2004-2005 9 months | A total of 162 children with febrile illness (88 and 74 in intervention and control groups respectively) were studied. Sixty-four (72.7 %) and 62 (83.8 %) of the febrile cases presumed to have malaria in the intervention and control groups respectively had parasitaemia on Day 0. The sensitivity and specificity of mothers’ diagnoses was 78.1 % and 29.2 %; 82.3 % and 8.3 %; in the intervention and control groups respectively. |
| Sule, 2009[97] | SW; State unspecified and conducted in two semi urban communities | Pre/Post-interventional quasi-experimental/ Controlled community based intervention | Mothers of children aged  0–18 months | 150 mothers of children aged 0–18 months selected independently from the intervention and control communities through a multi-stage sampling technique | Nutritional education to improve knowledge, attitude and practices (KAP) of mothers concerning infants and young children feeding | Nil | Unspecified | Year unspecified | Before intervention, recruited mothers and their children from the two communities were comparable in terms of all the parameters assessed (P > 0.05 in all cases). After six months of intervention, mothers who had nutritional education demonstrated better knowledge and attitudes to key infant and young children feeding recommendations. There was also limited improvement in feeding practices. Mothers from the intervention community exclusively breastfed their infants longer with mean age at introduction of complementary foods at 5.3 months compared to 4.5 months in the control community (P < 0.05), breastfed their children longer (P < 0.05). However, there was no statistically significant improvement in the weight of their children. |
| Chirdan, 2008 [68] | NC; Plateau;  Bassa; Rural | Pre/Post-interventional quasi-experimental/ Community based intervention | Caregivers of under-five children | Multistage sampling technique was used  to select caregivers from households with an under-five child; 150 caregivers were recruited | Health education on home treatment and prevention of malaria for caregivers of under-five children | Nil | University of Jos and Ahmadu Bello University Zaria | Unspecified | All the respondents recognized malaria as one of the diseases that cause fever in community. Sixty-one (40.6 %) had adequate knowledge concerning malaria causation, transmission, prevention and treatment. 28 (56 %) of respondents reported self-treatment. There was a statistically significant relationship between years of formal education and first line treatment option (P = .012). 34 (68 %) mothers acted within 8 hours of onset of fever. The intervention had an effect on perception (P < .001), knowledge (P < .001), malaria prevention practice (P = .001), first line treatment option (P = .031) and the type of treatment given to the children with fever (P = .048) |
| Okechukwu, 2008 [69] | NC; Abuja-FCT; Urban | Pre/Post-interventional quasi-experimental/Hospital based intervention | Exposed infants attending Paediatric Outpatient  Special  Treatment  Clinic (POSTC) of the  University of Abuja  Teaching  Hospital  (UATH) | The exposed infants were recruited and categorized based on their participation in the PMTCT programme. Deoxyribonucleic acid (DNA) Polymerase Chain Reaction (PCR) test was used for early diagnosis of HIV infection in the study infants. 160 HIV exposed infants were selected | Prevention of mother to Child Transmission of HIV programme | Exposed infants not participating in the programme | Government of Nigeria and unspecified foreign donor | Year unspecified 6 months | Overall transmission rate of HIV infection among the study subjects was 33.7 %. Transmission was found to occur in 6.7 % of infants who participated in PMTCT programme and in 68.6 % of those not involved in the programme, P < 0.001. For infants in the full programme, transmission occurred in 2.7 % of cases and in 25.0 % among those involved partially, P < 0.05 |
| Ajayi,2008 [51] | SW; Oyo;  Ona-Ara; Rural | Pre/Post-interventional quasi-experimental/ Community based intervention with control group | Caregivers of under-five children | The sample size for the survey was calculated  using com- pliance rate of 25 % for chloroquine when used at home to  treat febrile illness presumed to be malaria; 330 caregivers  participated in the study | Training a core group of mothers ("mother trainers") in selected communities  on the correct treatment of malaria and distributing a newly developed treatment guideline to each  household.  "Mother trainers" disseminated the  educational messages  about malaria and the  use of the guideline to their communities | Daily visits of study team to identify  children with febrile  ill- ness, ask questions about treatment practices and collect finger pricked blood sample for microscopy on Days 0, 1, 2, 3, 7 and 14. These visits were carried out for nine months. | University of Ibadan /University College Hospital, Ibadan | 2002-2004 13 months | Knowledge of cause, prevention and treatment of malaria increased with the one-year intervention. Many (70.4 %) of the respondents stated that they used the guideline each time a child was treated for malaria. There was a significant increase in the correct use of chloroquine from 2.6 % at baseline to 52.3 % after intervention among those who treated children at home in the intervention arm compared with 4.2 % to 12.7 % in the control arm. The correctness of use was significantly associated with use of the guideline. The timeliness of commencing treatment was significantly earlier in those who treated febrile children at home using chloroquine than those who took their children to the chemist or health facility (p < 0.005). Mothers considered the guideline to be explicit and useful. Mother trainers were also considered to be effective and acceptable. |
| Odusanya, 2003 [71] | SS; Edo; Sabongidda-Ora; Rural | Pre/Post-interventional quasi-experimental/ Community based intervention | Children aged  0–2 years and living in a rural community were recruited | A total of 327  children aged 0–2 years were recruited into the study | Privately financed immunization program to increase immunization coverage in a rural community | Nil | GlaxoSmithKline (formerly Mary Health Care Services such as health education and treatment of common childhood illnesses. SmithKline Beecham) Biologicals PLC | 1998-end point unspecified 2 years | Two years after the program was started, immunization coverage rates were 94 % for BCG, 88 % for DTP (third dose), and 82 % for measles. Antigens showed significant improvements from baseline values (p < 0.01). 84 % of children were fully immunized against six diseases, compared with 43 % at the commencement (p < 0.01). Hepatitis B coverage (three doses) was 58 %. |
| Amoran, 2013 [49] | SW; Ogun; Ijebu North; Rural | Pre/Post-interventional quasi-experimental/Community based intervention with a control group | Nursing mothers in the study area | A multistage  random sampling technique was  used in  choosing the  required sample of participants.  A total of 400 respondents were recruited into the study with 200 each in both the experimental and control groups | Health education intervention on malaria prevention practices  among nursing mothers  in rural communities | Baseline and post-intervention  assessment of knowledge and prevention practices of malaria | Olabisi Onabanjo University, Sagamu, Nigeria | Year unspecified 3 months | There was no statistically significant differences observed between the experimental and control groups. Knowledge of indoor spraying increased from 14.7 % to 58.2 % (P < 0.001) and window and door nets increased from 48.3 % to 74.8 % (P < 0.001). The proportion of those with ITN use increased from 50.8 % to 87.4 % (P < 0.001) while those with practice of maintaining clean environment also increased from 40.4 % to 54.5 % (P < 0.001). There were no significant changes in all the practice of malaria prevention methods in the control group. |
| Ukwaja, 2010 [93] | SW; Ogun; Abeokuta-south; Urban | Pilot pre/post-interventional quasi-experimental study | Under-five children resident in the catchment community of the study health facility | 50 eligible children were enrolled and treated with antibiotics with/out antimalarials based on rapid diagnostic test result | Malaria rapid diagnostic test-based management of childhood malaria-pneumonia | 50 children with  malaria-pneumonia symptom overlap were consecutively enrolled and treated presumptively with antibiotics and antimalarials irrespective of malaria test result | Government of Nigeria | 2009-2010; 3 months | The intervention and control arms did not differ significantly regarding patient demographic and clinical characteristics. Clinical cure rate was slightly higher in children managed presumptively 49 (98 %) than those managed rapid diagnostic test -based 47 (94 %) (P = 0.31). However, rapid diagnostic test -based treated children had lower risk of receiving antimalarials compared to those treated presumptively (48 % vs. 100 %), (P = <0.001; relative risk 2.08, CI 1.56 to 2.78). No death or severe complications were recorded in either group at day-5 follow-up |
| Kirwan, 2009 [94] | SW; Osun; Ile-Ife; Semi-urban | Double-blind placebo-controlled randomized trial | Children aged between one  and five years | Children aged  12–59 months were randomly  assigned to receive either albendazole or placebo every four months for 12 months with a follow-up at 14 months. Albendazole and placebo tablets were identical and manufactured by GlaxoSmithKline | Effectiveness of repeated four-monthly albendazole treatments on STH infection | Placebo | University of Dublin, Obafemi Awolowo University, Ile-Ife, Nigeria and HIV/AIDS Research and Policy Institute, Chicago State University, Chicago, IL, USA | 2006-2007; 1 year | 50 % of the preschool children in these semi-urban communities were infected by one or more helminths, the most prevalent STH being Ascaris lumbricoides (47.6 %). Our study demonstrated that repeated four-monthly anthelminthic treatments with albendazole were successful in reducing prevalence and intensity of A. lumbricoides infections. At the end of the follow-up period, 12 % and 43 % of the children were infected with A. lumbricoides and mean epg was 117 (S. E. 50) and 1740 (S. E. 291) in the treatment and placebo groups respectively compared to 45 % and 45 % of the children being infected with Ascaris and mean epg being 1095 (S. E. 237) and 1126 (S. E. 182) in the treatment and placebo group respectively at baseline |
| Ajayi, 2008 [98] | SW; Oyo; Ona-ara LGA; Ojoku/Ajia; Rural | Pre/Post Interventional study | Febrile children between six and 59 months of age reporting to trained community medicine distributors (CMDs) for microscopy and PCR analysis | Over a period of 12 months, a total of  432 children presenting with  fever were enrolled across the study sites | Presumptive antimalarial treatment with ACT- artemether-lumefantrine. Thick blood smears and blood spotted filter paper were prepared from finger prick blood samples collected for microscopy and PCR analysis | Nil | University of Ibadan, Nigeria; KNUST, Kumasi, Ghana; Makerere University, Kampala, Uganda; Ifakara Health Institute, Dar es Salaam, Tanzania | 1 year | Patent parasitaemia at baseline was present in 306 (70.8 %) C.I. (67 %–75 %). At day 28 it was 184 (60.1 %), crude parasitological failure was 77/184 (41.8 %) C.I. (35 %–49 %). Geometric mean parasite density at 28 days 2835. PCR adjusted failure rate = 14/154 (9.1 %) and PCR adjusted cure rate 90.9 % C.I. 86 %–95 % |
| Davies-Adetugbo,  1997 [102] | SW; Osun; Ife  south and Atakumosa LGAs; Rural | Community based pre/post-interventional Intervention study with control group | Eligible health workers in the study area consisting of  ten health districts, with a total of 30  health facilities | 30 PHC workers attended TOT. A further 36 PHC workers and 569 community members received training at these district-level workshops | Training of community health extension workers  to promote exclusive breastfeeding in rural communities | No intervention was conducted | Obafemi Awolowo University, lie- Ife | Unspecified | In the study area perinatal facilities, early initiation of breastfeeding has increased compared with those in the control area (P < 0.001). Also, the trained health workers had significantly better knowledge about breastfeeding than their untrained colleagues in both the study (P < 0.001) and control areas (P < 0.001), and more often recommended timely initiation and exclusive breastfeeding than the controls (P < 0.001). A multivariate analysis showed that the training programme and the study area were the only significant variables that were predictors of breastfeeding knowledge (P < 0.001) |
| Molta, 1992 [107] | NE | Community based  drug efficacy trial | A total of 2056 children under 5 years screened  for malaria infection | 170 (14.3 %) of  these positive  children were  enrolled into the  study | Demonstrating the clinical and parasitologic efficacies of oral chloroquine phosphate, pyrimethamine/sulphadoxine and pyrimethamine/sulphalene in treating Plasmodium falciparum malaria | Nil | University of Maiduguri | 1988-1990;  2 years | Clinically, the chloroquine phosphate demonstrated high performance in clearing symptoms of infection. However, varying degrees of parasitologic failure, ranging from delayed clearance through recrudescence to asymptomatic Type-II resistance, were encountered. For tests with pyrimethamine/sulphadoxine and pyrimethamine/sulphalene, 517 and 253 children, respectively, were screened. The corresponding infection rates were 71.6 % (370 children) and 71.5 % (181 children), with 59 and 34 enrollments. Both drugs were highly effective, clinically and parasitologically |
| Oche, 2013 [73] | NW; Sokoto; Wamakko; Rural | Pre/Post-interventional quasi-experimental study/community  based | Mothers with at least a child or children aged 6 months to 6 years were eligible for the study. | There are 312  houses in Garabshi with a total  population of  women of childbearing age of 170. Using systematic sampling method, a one in three samples of fifty mothers was recruited for the study. In a house with no woman of child bearing age with eligible children, the next house was chosen and since polygamy is well entrenched in the community, in a house with more than one woman of child bearing age, one was chosen by using simple random sampling using a toss of coin. | Health education to  improve knowledge and home management of febrile convulsion amongst mothers in a rural community | Nil | Usmanu Danfodiyo University, Sokoto = [ | Unspecified | The perceived causes of febrile convulsion included fever (28 %), witch craft (80 %) with majority (98 %) of the mothers administering traditional medications. Proportion of study subjects with adequate knowledge of febrile convulsion at baseline and post intervention were 4 % (mean knowledge score of 35.3 ± 9.48) and 96.0 % (mean knowledge score of 77.69 ± 10.75) respectively (P < 0.0001) |
| Amoran, 2012 [55] | SW, Ogun, Ijebu North LGA, rural | Pre/Post community based Intervention  design with a control group | Mothers or guardians of children less  than fives | Only mothers or guardians who were permanent residents (resident in the area >6 months) and currently having children under 5 living with them were included in the study | Structured educational programme based on a course content adapted  from the National Malaria Control Programme | Baseline and post-intervention  assessement of ITN  use | Olabisi Onabanjo University, Sagamu, Nigeria | 2010 3 months | The ITN ever users in experimental group were 59 [29.5 %] and 138 [72.6 %] in pre and post intervention period, respectively (p value =0.001). These proportions of ITN ever users were 55 [27.5 %] and 57 [31.6 %] in control group, during the pre and post intervention periods (p = 0.37). Post health education intervention, degree of change in knowledge of ITN re-treatment [37.0 %] and mounting [33.5 %], readiness to use if given free [30.5 %] and belief in efficacy [36.9 %] improved significantly in the experimental group while there was no significant change in the control group [p = 0.84, 0.51, 0.68 & 0.69 respectively |
| Integrated maternal, newborn, and child health (crosscutting community and health system strategies) interventions | | | | | | | | | |
| Okafor, 2011 [30] | SE, Enugu,  Enugu, Urban | Pre/Post-interventional quasi-experimental/Hospital based intervention | All pregnant women and neonates  residing in the catchment area  of the intervention hospital | All pregnant  women and  neonates in their  first week of life  that attended clinic within the period of study | Introduction of Free Maternal and Child Health Care (FMCHC) in Enugu State University Teaching Hospital, SE Nigeria | Nil | Government of Nigeria | 2008 6 months | Uptakes of antenatal booking (202.2 %), and hospital delivery (151.8 %). It also resulted in decreased maternal and perinatal mortality by 16.4 % and 34 %, respectively |
| Adinma, 2011 [58] | SE, Anambra, Igboukwu and Ekwuluobia,  Two rural communities | Pre/Post-interventional quasi-experimental/ Community based intervention with  control arm | Females of reproductive age group (15–49 years) each  with at least one under five-year old child, from each of the study communities. | The WHO Cluster Sampling  Technique was  used in this  study to select the required 120 participants  from each of the intervention and control communities. | Introduction of a Government-Community Healthcare Co-Financing  on Maternal and Child Healthcare in Nigeria | Baseline and post-intervention assessment of utilization of MNCH services | Government of Nigeria | 2004 1 year | Better utilization of health services occurred in the intervention area post-intervention. Quality of service from intervention clients’ perspective showed significant improvement post-intervention. Distance less than 5 km, transportation cost less than N40 (about US$0.25), and maternal education above secondary level impacted positively on utilization of maternal and child health services. Acceptability of the scheme was better for the intervention facility evident from the higher number of respondents showing “willingness to join”, and “willingness to pay”. |
| Adinma, 2011 [58] | SE, Anambra, Aguata, Rural | Pre/Post-interventional quasi-experimental/ Community based intervention | Women of child bearing age residing in the study area | Selection process unspecified; 120 women of reproductive age  were selected | Integrating maternal  health services into a  health insurance scheme to improve healthcare delivery | Nil | Government of Nigeria | Unspecified | Utilization of maternal health services % antenatal and delivery services, were significantly better at the late intervention period when compared to the early intervention period. Quality of service from clients' perspective also showed significant improvement at the late intervention period. There was an overall greater availability of maternal health service equipment, drugs and consumables, and medical records in the health facility later during the scheme |
| Thompson A. 2010  [63] | NW; Kaduna; Kaduna North; Urban | Pre/Post-interventional quasi experimental/Hospital based Intervention | Facility staff  and clients attending the  Demonstration Clinic for the Family Health Unit (Asibitin Yara) in the Tudun  Wada area of Kaduna | Between 60–100 clients and patients visited the clinic  each week day and have their patient records created and updated without hindering or slowing patient care | Pilot demonstration of the software (OpenMRS) to Support Maternal and Reproductive Health information management system |  | University of California Santa Cruz and Shehu Idris College for Health Sciences and Technology | 2009 3 months | The initial reaction by the staff to OpenMRS was very positive. Most people at the clinic had never used a computer before but all claimed to be willing to learn. The people with past computer experience were more interested in learning OpenMRS. Initially, training began with the two men in the Records Department, both who had previous experience with computers. They were able to understand OpenMRS and navigate the patient creation, patient look up and encounter form entry after only hours of introduction. The problems encountered while teaching were: language barrier, spelling errors, typing speed, and overall speed on computer. The clinic handles a large volume of clients each day and the overall speed of the two Records Keepers was not fast enough to record all the patients every day. |
| Okonofua, 2010 [20] | Whole country; Urban and rural | Pre/Post-interventionalquasi-experimental | The three tier system of government of Nigeria  consisting of  the Federal Government, 36 States including the Federal Capital Territory (FCT), and 774 Local Government Councils | The chief executives and policy makers were targeted; the  36 state governors  or their representatives  were interviewed  and targeted for advocacy | Advocacy program (advocacy and policy education) aimed at implementing a policy of free maternal and child health (MCH) services in Nigeria | Nil | Government of Nigeria | 2006; 3 years | At baseline in December 2006 the States offering free treatment for pregnant women and under 5 children - n = 4 (10.8 %); Nasarawa, Balyesa, Taraba, Osun States with partial coverage of free treatment for pregnant women and children-n = 11 (29.7 %); Rivers, Gombe, Kano, Jigawa, Anambra, Ogun, Ondo, Lagos, Ebonyi, Zamfara, Kebbi States not offering free medical services - n = 22 (59.5 %); Borno, Adamawa, Plateau, Katsina, Bauchi, Cross River, Niger, Edo, Ekiti, Sokoto, Oyo, Delta, Kwara, Imo, Kogi, Benue, Yobe, Abia, Enugu, FCT, Akwa Ibom, Kaduna The number of States offering comprehensive free MCH services increased from four to nine; the States offering partially free MCH services increased from 11 to 14 (8.1 % increase); while those not offering any form of free treatment decreased from 22 to 14 (21.7 % decrease). |
| Findley, 2013 [74] | NE and NW; Katsina, Yobe  and Zamfara;  Urban and rural | Quasi-Experimental/ Community Based Intervention with  control group | Women of child bearing age and under-five children | Stratified cluster sample household surveys was conducted at  baseline (2009)  and follow-up  (2011) to assess changes in newborn and sick child care practices among women with births in the five prior years (baseline: n = 6,906; follow-up: n = 2,310) | Community-based  approach to promoting improved newborn and  sick child  care through community volunteers and Community Health Worker | Baseline (2009) and follow-up (2011) to assess changes in newborn and sick child care practices | DFID and the  Norwegian  Government | 2009-2011 2 years | Anti-tetanus vaccination coverage during pregnancy increased from 69.2 % at baseline to 85.7 % at follow-up in the intervention areas. Breastfeeding within 24 hours increased from 42.9 % to 59.0 % in the intervention areas, and more newborns were checked by health workers within 48 hours (from 16.8 % at baseline to 26.8 % at follow-up in the intervention areas). Newborns were more likely to be checked by trained health personnel, and they received more comprehensive newborn care. Compared to the control communities, more than twice as many women in intervention communities knew to watch for specific newborn danger signs. Compared to the control and low-intensity intervention communities, more mothers in the high-intensity communities learned about the care of sick children from CHWs, with a corresponding decline those seeking advice from family or friends or traditional birth attendants. Significantly fewer mothers did nothing when their child was sick. High-intensity intervention communities experienced the most decline. Those who did nothing for children with fever or cough declined from 35 % to 30 %, and with diarrhea from 40 % to 31 %. Use of medications, both traditional and modern, increased from baseline to follow-up, with no differentiation in use by intervention area. |
| Ashir, 2012 [73] | NE; Yobe;  Geidam; Rural | Pre/Post-interventional quasi-experimental/Community based intervention with a control group | Women of child bearing age | The total population of the PBF intervention communities was projected to be  30,000 according to the 2006 population census. The target population for the PBF incentive was 7,500 (women and children) | Piloting of Performance Based Financing and  Uptake of Maternal and Child Health (MCH) Services | Baseline and post-intervention  assessment of the utilization of MCH services | DFID and the  Norwegian  Government | 2011--end  point unspecified 6 months | The demand-side PBF led to increased utilization of key MCH services (antenatal care and skilled delivery) but had no significant effect on completion of child immunization using measles as a proxy indicator |
| Findley, 2013 [22] | NE and NW; Katsina,  Yobe and Zamfara; Urban and rural | Pre/Post-interventional quasi-experimental/ Community based intervention with  control group | Women of child bearing age and under-five children | Stratified cluster sample household surveys was conducted at baseline (2009) and follow-up (2011) to assess changes in newborn and sick child care practices among women with births in the five prior years (baseline: n = 6,906; follow-up: n = 2,310) | Integrated maternal, newborn, and child health program, Northern Nigeria | Baseline and post-intervention  assessment of the MNCH outcomes | DFID and the  Norwegian  Government | 2009-2013;  4 years | Between baseline and follow-up, anti-tetanus vaccination rates increased from 69.0 % to 85.0 %, and early breastfeeding also increased, from 42.9 % to 57.5 %. More newborns were checked by trained health workers (39.2 % to 75.5 %), and women were performing more of the critical newborn care activities at follow-up. Fewer women relied on the traditional birth attendant for health advice (48.4 % to 11.0 %, with corresponding increases in advice from trained health workers. At follow-up, most of these improvements were greater in the intervention than control communities. In the intervention communities, there was less use of anti-malarials for all symptoms, coupled with more use of other medications and traditional, herbal remedies. Infant and child mortality declined in both intervention and control communities, with the greatest declines in intervention communities. In the intervention communities, infant mortality rate declined from 90 at baseline to 59 at follow-up, while child mortality declined from 160 to 84. |
| Pathfinder, 2011 [84] | NE, NW and SW; Kano, Lagos, and Borno; Minjibir, Agbowa and Gwoza; Rural | Intervention process  and output evaluation | Community and political leaders. Facility health workers (FHW) and community health workers (CHW) | In the intervention areas there was selection and  targeting of intervention on ommunity and political leaders. Facility health workers (FHW) and community health workers (CHW) | Maternal Health Care Improvement Initiative framework employs community capacity building and health system strengthening approaches to address the community and health system roots of delays in delivering maternal care | Nil | Government of Nigeria, MacArthur Foundation and Pathfinder | 2003-2010;  7 years | 62 MCHIC members trained in maternal health core concepts and advocacy: 20 in Gwoza; 22 in Minjibir; 20 in Agbowa. • 30 facility health workers re-trained in MH care service delivery. • 60 male motivators and 60 young mother peer educators trained in MH core concepts and behavior change communication. • 740 FHWs, CHWs, TBAs, male motivators, peer educators, community leaders, and government representatives trained in referral linkage systems. • Observed increase in community service uptake for skilled birth attendants. • Established community structures for maternal health service improvement monitoring. • 144 supervisory MCHIC visits to facility sites and monitoring of CHW rounds. |
| Adeleye, 2011 [92] | SS; Edo; Ovia North-East; urban | Intervention process  and output evaluation | All adult males resident in the study area | 31-member Elders’ Council and  members of the young adults  members of the  youth association. Participants were recruited through locally accepted ways of communication and modes of authority | Communication  intervention (Group health talks) to improve male attitudes and practices regarding their involvement in prenatal care and family planning | Nil | Government of Nigeria and unspecified collaborator | 4 weeks | Using gender theory as an analytical lens along with the application of local cultural beliefs and norms, a useful communication intervention was developed that increased the possibility of positive male engagement in maternal health in 1 Nigerian community |
| Mojidi, 1998 [101] | International study reporting on Nigeria; Local intervention area unspecified | Post-intervention  impact evaluation | Hardest to reach and the most underserved groups | Women of child bearing age and their spouses resident in  the study area | Expand access to new sources of information and knowledge within traditional communities and societies. Involving men in programs initially targeted to expand family planning to women | Nil | CEPDA/ACCESS  project | Unspecified | First, that the gender sensitivity decreases male resistance to family planning. Second, that linking rural health and development initiatives motivates men to seek treatment and encourages condom use, and third, building strategic alliances with male groups elevates women's family planning/rural health/maternal-child health issues and encourages male support for partner family planning/rural health decisions |
| Brieger, 1997 [104] | NW and SW;  Kano and Lagos; Kano city, Lagos Island, Ajegunle, Amukoko, Mushin, Lawanson, and Makoko; Urban | Process evaluation | Community -based organizations (CBOs) and  health facilities  in intervention area | Community Partnerships for Health (CPH)  Board members | CPH programs for the formation of local partnerships or coalitions for promoting, planning, and delivering child and family health services | Nil | Community  Partnerships for Health (CPH) programs | Sept-Dec 1997; 3 months | Memberships came mostly from CBOs. 33 % of CPH Board members were women. CPHs rated themselves strongest on establishment of an organizational structure and weakest in the area of programming. CPHs relied on collection of dues and donations, fund-raising activities, and income generation projects for financial sustainability. CPHs are accessing resources and technical assistance from governmental and nongovernmental organizations and are communicating between each other for assistance and advice on starting new projects and solving problems. CPHs in Kano appear to be able to rely on medicine vendors and indigenous healers for adopting the CPH approach requiring a guarantee of affordable and timely care to prevent death from childhood diseases. Kano CPH Board members include only 5 % who are female. |
| Galadanci, 2010 [108] | NW; Kano; Urban and rural | Impact evaluation | Women of child bearing age resident in the study area | Cohort of women  who used ANC services and gave birth during the period of intervention | Introduction of free maternity services | Nil | Aminu Kano Teaching Hospital/Bayero University Kano, Pathfinder International,Government of Nigeria | 2001-2006;  5 years | Since the introduction of free maternity services in 2001, ANC attendance from 28 hospitals increased from 303,649 in 2001 to 705,468 in 2006. Deliveries increased from 29,704 in 2001 to 42,127 in 2006. In one hospital, caesarean section rate increased from 2.82 % in 2000 to 8.12 % in 2005 |

Geographical location is classified as NC, NE, NW, SE, SS and SW or State and Local Government Areas (LGA).

*NC = North central, NE = North east, NW = North west, SE = South east, SS = South south and SW = South west (regions of the country).

**CYP = couple years of protection***EmOC = Emergency obstetrics care****YLS = Years of life saved *****MIP = Malaria in p ******CORP = Community oriented resource persons ******* NASG = Non Pneumat.
